# The VQ motif-containing proteins in the diploid and octoploid strawberry

**DOI:** 10.1038/s41598-019-41210-4

**Published:** 2019-03-20

**Authors:** José Garrido-Gala, José Javier Higuera, Juan Muñoz-Blanco, Francisco Amil-Ruiz, José L. Caballero

**Affiliations:** 1 Departamento de Bioquímica y Biología Molecular, Edificio Severo Ochoa-C6, Campus Universitario de Rabanales y Campus de Excelencia Internacional Agroalimentario ceiA3, Universidad de Córdoba, 14071 Córdoba, Spain; 2 Unidad de Bioinformática, Servicio Central de Apoyo a la Investigación (SCAI), Universidad de Córdoba, 14071 Córdoba, Spain

## Abstract

The plant VQ motif-containing proteins are a recently discovered class of plant regulatory proteins interacting with WRKY transcription factors capable of modulate their activity as transcriptional regulators. The short VQ motif (FxxhVQxhTG) is the main element in the WRKY-VQ interaction, whereas a newly identified variable upstream amino acid motif appears to be determinant for the WRKY specificity. The VQ family has been studied in several species and seems to play important roles in a variety of biological processes, including response to biotic and abiotic stresses. Here, we present a systematic study of the VQ family in both diploid (*Fragaria vesca*) and octoploid (*Fragaria x ananassa*) strawberry species. Thus, twenty-five VQ-encoding genes were identified and twenty-three were further confirmed by gene expression analysis in different tissues and fruit ripening stages. Their expression profiles were also studied in *F*. *ananassa* fruits affected by anthracnose, caused by the ascomycete fungus *Colletotrichum*, a major pathogen of strawberry, and in response to the phytohormones salicylic acid and methyl-jasmonate, which are well established as central stress signals to regulate defence responses to pathogens. This comprehensive analysis sheds light for a better understanding of putative implications of members of the VQ family in the defence mechanisms against this major pathogen in strawberry.

## Introduction

Plant growth and development are constantly affected by changing environmental conditions and stresses in both, natural and agricultural settings^1^. Among the most important biotic stresses are those caused by microbial pathogens like viruses, bacteria and fungi. Thus, plants have evolved various and complex defence systems to protect themselves from pathogens, which are finely regulated by a large and diverse set of regulatory proteins including transcription factors (TFs), that bind to specific *cis*-regulatory elements in the promoter region of target genes controlling their transcription^2^.

Several TF families are particularly involved in regulating the defence responses in plants: AP2/ARF, bHLH, bZip, MYB, NAC and WRKY^3^. WRKY TFs are one of the largest families of transcriptional regulators existing in plants. They are structurally characterized by a highly conserved DNA binding domain, about 60 amino acids long, harbouring one or two core motif WRKYGQK and a Zinc-finger-like motif with two variants: C2H2 or C2HC. WRKY proteins are classified in Groups I, II and III on the basis of both the number of WRKYGQK motifs and the features of their Zinc-finger motif. The polyphyletic Group II is further divided into subgroups IIa, IIb, IIc, IId and IIe based on differences in their amino acid sequence^4–6^. WRKY TFs bind to target gene *cis*-elements with sequence TTGAC[C/T] known as W-box. They have been referred as the “jack of many trades in plants”, forming an intricate network that play diverse roles in regulating the transcriptional activity of plant cells to execute several developmental programmes and stress responses^7^.

Regulatory proteins that do not bind DNA directly can form protein-protein complexes with TFs to fine-tune the transcriptional response. A recently identified group of proteins containing a short and conserved amino acid motif, named VQ proteins, belongs to this class. VQ motif-containing proteins have been characterized in a number of plants including Arabidopsis with 34 VQ members^8^, rice with 40 VQ members^9^, soybean with 74 VQ members^10^, and grapevine with 18 VQ members^11^. Very recently, an *in silico* analysis of the VQ protein family addressing the phylogenetic relationships and microevolution of VQ genes in the genus *Fragaria* was published^12^. Low sequence similarity has been found between known plant VQ proteins and proteins from other organism, suggesting that the VQ family is highly specific to plants^13^. However, in a more recent phylogenetic study, some non-plant proteins containing partial or divergent VQ motifs were found^14^. Plant VQs are relatively short in length and mostly coded by intronless genes but two or more introns have also been found^15^. All plant VQs share the conserved amino acid motif FxxhVQxhTG involved in protein binding with several WRKY TFs but they are variable in length and highly divergent in amino acid sequence outside of the VQ domain^8^.

A number of evidences have shown that plant VQ proteins bind to the C-terminal WRKY domain of Group I and to the single one of Group IIc WRKY proteins. Lai *et al*.^17^ demonstrated that the conserved V and Q residues in the VQ motif of SIB1 (VQ23) are essential for the interaction with WRKY33 in *Arabidopsis* and proposed the VQ motif as the core of the WRKY-interacting motif^16^. Recently, Zhou *et al*.^10^ identified an additional eight amino acids long upstream motif close to the VQ motif as an important region for the affinity and specificity of WRKY-VQ binding. Thus, single amino acid substitutions changed the specificity patterns for their WRKY target in the mutated VQs while did not abolish their binding to WRKY proteins, except by deletion of the whole motif in GmVQ22^10^. A very recent work in apple confirms that the amino acid residues flanking the core VQ motif are also required for the interaction with the WRKY domain, as well as the specificity of the VQ proteins for the Group I and IIc of WRKY TF^17^.

VQ proteins can activate or repress transcriptional activity of Group I and IIc WRKY proteins^13^. *Arabidopsis* VQ23 and VQ16 (SIB1 and SIB2, respectively) recognize the C-terminal WRKY domain of WRKY33 and stimulate its DNA binding activity, thus playing a positive role in defence against necrotrophs. Indeed, WRKY33, SIB1 and SIB2 were markedly induced by *B*. *cinerea* infection and showed similar expression patterns^16^. *Arabidopsis* VQ29 can interact with WRKY25 and WRKY33^8^ but also with PIF1, a bHLH TF that negatively regulates light-dependent seed germination^15^. While it positively stimulates the activity of PIF1 (hence, acting as repressor of seedling de-etiolation), its overexpression increases susceptibility to *B*. *cinerea* in *Arabidopsis* leaves and thus, acting as a negative regulator of the defence response^18^. In contrast, *AtVQ29* expression is induced early upon infection in *Arabidopsis* roots by *P*. *parasitica* and it is required to restrict the pathogen development independently of SA-, JA- and ET-mediated defence activation^19^. Thus, AtVQ29 can modulate different responses either positively or negatively, depending of the tissue and stimulus. On the other hand, MKS1 (AtVQ21) forms complexes with MPK4 and WRKY33. After pathogen infection, phosphorylation by MPK4 occurs and the MKS1-WRKY33 complex is activated and can dissociate, promoting the *PAD3* expression and camalexin biosynthesis. Thus, MKS1 was shown to be acting as repressor in absence of *P*. *syringae* infection or flagelin treatment^20^. Similarly to the MKS1-MPK4 interaction, other VQ proteins have been proven as substrates for the mitogen-activated protein kinases MPK3 and MPK6. Phosphorylation of a subset of VQ proteins by MPK3/6 destabilizes their association with WRKY proteins, leading to their release from the complex. This can promote direct WRKY-DNA binding or alternatively, the substitution for a different VQ protein, thus regulating transcriptional activity upon PAMP recognition and providing an additional and finely regulated mechanism for transcriptional plant defence control^21,22^.

The genus *Fragaria* (*Rosaceae*) comprises about 24 species worldwide distributed, including diploids and polyploids, wild and cultivated members^23^. The octoploid cultivated strawberry (*Fragaria x ananassa* Duch.) is an important and appreciated fruit crop with valuable organoleptic and nutritional qualities^24^. With an annually increasing, world production of more than 9 Mt and 401,862 ha cultivated (FAOSTAT, 2016) it is presumably, the most economically important soft berry.

For the past several years, continuous efforts to dissect the genomic structure and genetic regulation of this valuable crop are being made, which is an inescapable requisite for both, basic and applied research, in aspects such as identification of genetic markers linked to valuable traits for breeding, improving fruit quality, stress response regulation or comparative genomics. The diploid *Fragaria vesca* genome was firstly sequenced and published in 2011^25^ and recently re-sequenced *de novo*^26^. The first assembly versions have been later improved^27^ and re-annotated subsequently with RNA-seq support^28,29^. The genomes of cultivated strawberry (*Fragaria* x ananassa) and four wild *Fragaria* species, representing the genetic diversity into the genus (*F*. *nipponica*, *F*. *iinumae*, *F*. *orientalis*, and *F*. *nubicola*) were sequenced and a reference genome of *Fragaria* x *ananassa* was partially assembled and designated as FANhybrid_r1.2^30^. One remarkable result was the high sequence similarity with *F*. *vesca* (assembly version 1.1), with only 5.23% of non-homologous sequences. Besides the previously observed high levels of conserved macrosynteny and colinearity between the diploid and octoploid *Fragaria* genomes, this find strengthen support for the use of the former as model for genomic research in the latter, much more complex^31^.

Strawberry anthracnose is a severe disease caused by the fungus *Colletotrichum*, one of the most important genera of strawberry pathogens. *Colletotrichum acutatum*, *Colletotrichum fragariae* and *Colletotrichum gloeosporioides* are the three major species causing the fruit and crown rot diseases in strawberry^32^. *Colletotrichum spp*. is a hemibiotrophic pathogen, switching from a short, symptomless biotrophic stage to a necrotrophic phase, characterized by host cell death and extensive tissue colonization, producing the typical anthracnose symptoms. In the past years, a few transcriptomic studies focused in the *Colletotrichum*-strawberry interaction have been conducted^33–35^ with no specific findings about possible roles of the VQ protein family in the defence mechanisms of strawberry to this pathogen.

In the present work, a deep and systematic analysis of the strawberry VQ proteins was performed using the latest available, annotated reference genomes and transcriptomes of both, diploid and octoploid species. We investigated the phylogenetic relationships, conserved domains and structure among four representative species, in an effort to establish a more robust classification of the VQ family into functional groups. As a remarkable result of the domains screening, we also describe a novel protein domain association and propose the name of R protein-VQ for the VQ proteins harbouring NBS-ARC and LRR-like domains, typical of R proteins. In addition, in order to elucidate putative roles of members of the VQ protein family in the defence mechanisms of strawberry against pathogens, the expression pattern of the VQ genes (*Fragaria* x ananassa cv. Camarosa) were analysed in different plant tissues and fruit ripening stages as well as in response to fruit infection with *C*. *acutatum* and salicylic acid and methyl-jasmonate treatments.

## Results and Discussion

### VQ members of *F*. *vesca* and *F*. *ananassa*

**Figure 1:**
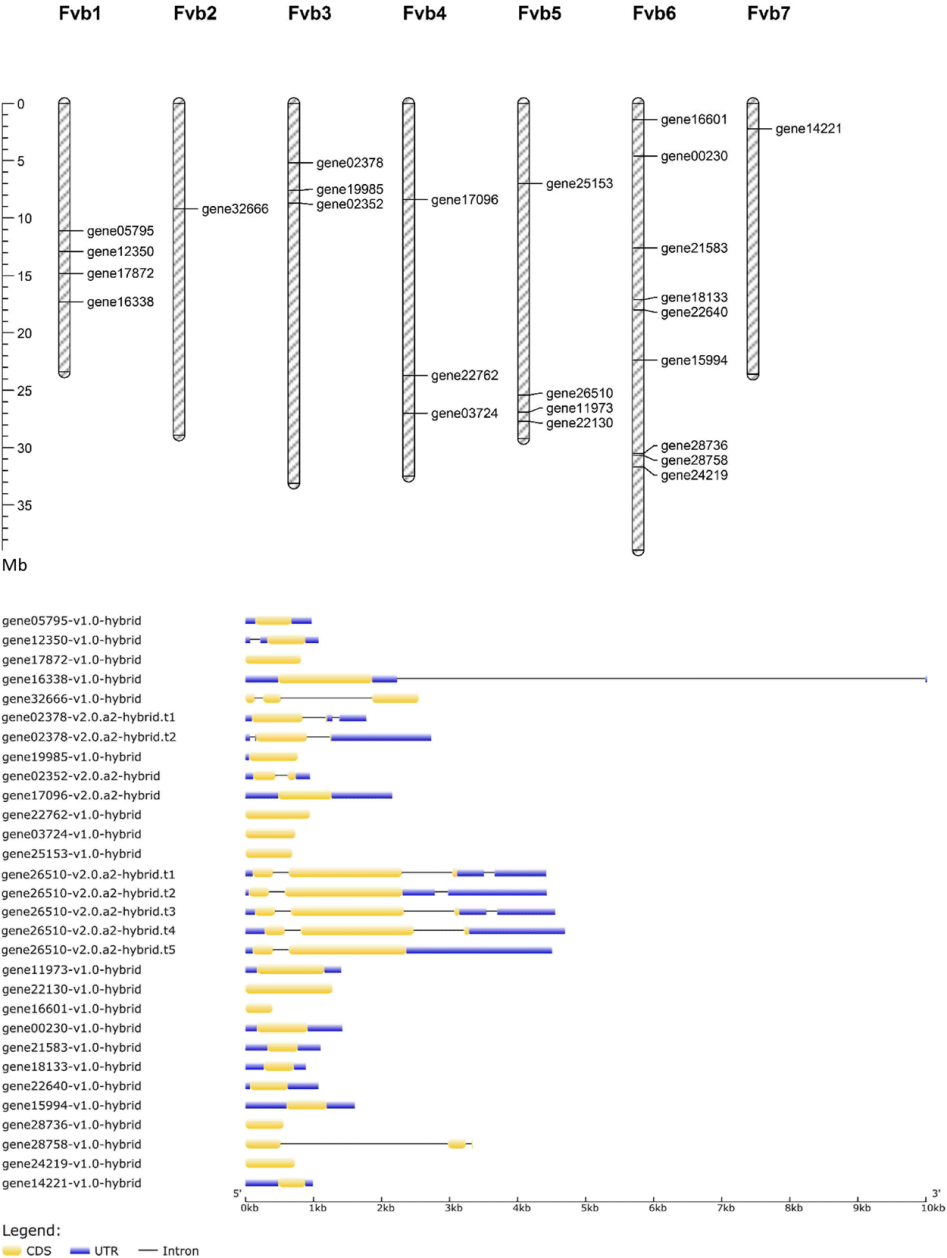
Chromosome mapping and gene structure of the *Fragaria vesca VQ* gene family.

Characteristics of *FvVQ* genes found in the genome annotation of *F*. *vesca* v2.0.a2 and their corresponding predicted proteins are listed in Table 1 and named *FvVQ1* to *FvVQ25* according to their chromosomal order. Most of the *FvVQ* genes are relatively short, intronless (18 out of 25) and they are unevenly distributed in the genome, being the chromosome 6, which contains the higher number of them (Fig. 1). As it has been previously described for other species^8,10,11^, most of the strawberry *FvVQ* genes encode short proteins ranging from 131 to 678 aa, with an average of 274. Also, two and five alternative RNA splicing forms were found for *genes FvVQ6* and *FvVQ13*, respectively. Furthermore, as expected, most of the FvVQ proteins are predicted to locate in the nucleus and show monopartite or bipartite nuclear location signals, while FvVQ1 and FvVQ3 also contain putative chloroplast import signals. Despite of the absence of classical NLSs in some FvVQ, their predictions agree with previous evidence that VQ proteins can be located in the nucleus, where they interact with WRKY TFs to modulate the gene expression^36^.

**Table 1:** List of the strawberry VQ members and selected properties. Subcellular locations predicted by LOCALIZER are: ch, chloroplast; n, nucleus. Nuclear location signals (NLS) predicted by SeqNLS with scores greater than 0.5 are marked by asterisk. Note that some of the isoforms present identical or similar protein properties.

Name	Gene id. (*F*. *vesca* v2.0.a2)	Genomic location	Splicing forms	Exons	Protein properties				
					Length (aa)	MW (KDa)	PI	Domains	Subcellular location
*FvVQ1*	*gene05795-v1*.*0-hybrid*	Fvb1:11109731.0.11110700 (+strand)		1	177	19.40	9.58	VQ	ch*
*FvVQ2*	*gene12350-v1*.*0-hybrid*	Fvb1:12947350.0.12948422 (+strand)		2	186	20.69	9.55	VQ	
*FvVQ3*	*gene17872-v1*.*0-hybrid*	Fvb1:14815114.0.14815929 (−strand)		1	271	29.32	8.10	VQ	ch/n
*FvVQ4*	*gene16338-v1*.*0-hybrid*	Fvb1:17333502.0.17343519 (−strand)		2	460	49.20	6.67	VQ	n*
*FvVQ5*	*gene32666-v1*.*0-hybrid*	Fvb2:9213921.0.9216468 (+strand)		3	361	39.48	6.87	VQ	n*
*FvVQ6*	*gene02378-v2*.*0*.*a2-hybrid*	Fvb3:5205231.0.5207960 (−strand)	t1, t2	3	254	27.71	9.76	VQ	*
*FvVQ7*	*gene19985-v1*.*0-hybrid*	Fvb3:7601133.0.7601903 (+strand)		1	238	26.20	5.75	VQ	*
*FvVQ8*	*gene02352-v2*.*0*.*a2-hybrid*	Fvb3:8749508.0.8750454 (+strand)		2	148	16.35	10.14	VQ	
*FvVQ9*	*gene17096-v2*.*0*.*a2-hybrid*	Fvb4:8384711.0.8386867 (+strand)		1	261	28.78	9.58	VQ	
*FvVQ10*	*gene22762-v1*.*0-hybrid*	Fvb4:23658011.0.23658958 (+strand)		1	315	33.98	10.80	VQ	
*FvVQ11*	*gene03724-v1*.*0-hybrid*	Fvb4:27000140.0.27000874 (−strand)		1	244	27.19	6.82	VQ	
*FvVQ12*	*gene25153-v1*.*0-hybrid*	Fvb5:6972449.0.6973138 (−strand)		1	229	24.75	5.83	VQ	n*
*FvVQ13*	*gene26510-v2*.*0*.*a2-hybrid*	Fvb5:25430959.0.25435652 (+strand)	t5	2	676	77.38	7.53	VQ, LRR8, NB-ARC	
			t2	3	676	77.39	7.54		
			t4	3	678	77.27	6.89		
			t1,t3	4	678	77.28	6.90		
*FvVQ14*	*gene11973-v1*.*0-hybrid*	Fvb5:26904474.0.26905877 (+strand)		1	330	35.67	10.05	VQ	*
*FvVQ15*	*gene22130-v1*.*0-hybrid*	Fvb5:27727944.0.27729224 (−strand)		1	426	46.02	7.02	VQ	n*
*FvVQ16*	*gene16601-v1*.*0-hybrid*	Fvb6:1405916.0.1406311 (+strand)		1	131	15.15	5.56	VQ	n**
*FvVQ17*	*gene00230-v1*.*0-hybrid*	Fvb6:4590905.0.4592326 (+strand)		1	248	26.40	9.47	VQ	n*
*FvVQ18*	*gene21583-v1*.*0-hybrid*	Fvb6:12584223.0.12585325 (+strand)		1	149	16.35	6.05	VQ	n*
*FvVQ19*	*gene18133-v1*.*0-hybrid*	Fvb6:17070375.0.17071259 (+strand)		1	148	16.56	9.25	VQ	n*
*FvVQ20*	*gene22640-v1*.*0-hybrid*	Fvb6:18023651.0.18024721 (−strand)		1	184	20.68	9.27	VQ	n*
*FvVQ21*	*gene15994-v1*.*0-hybrid*	Fvb6:22449818.0.22451424 (+strand)		1	195	21.36	8.03	VQ	
*FvVQ22*	*gene28736-v1*.*0-hybrid*	Fvb6:30523488.0.30524048 (+strand)		1	186	20.78	4.30	VQ	n
*FvVQ23*	*gene28758-v1*.*0-hybrid*	Fvb6:30654008.0.30657346 (−strand)		3	263	29.62	4.77	VQ	n *
*FvVQ24*	*gene24219-v1*.*0-hybrid*	Fvb6:31699569.0.31700294 (+strand)		1	241	25.53	10.24	VQ	n*
*FvVQ25*	*gene14221-v1*.*0-hybrid*	Fvb7:2184725.0.2185715 (+strand)		1	132	15.20	7.31	VQ	n*

BLASTN searches with the *FvVQ* set as query were performed to find the *Fragaria* x ananassa orthologous *VQ* genes (*FaVQ*) and transcripts from both the reference genome and RefTrans V1 RNA-seq annotation. Results were compared with those obtained by the HMM3 searches for both datasets. Thus, many orthologous sequences were found matching with the RefTrans V1 transcripts and the FANhybrid scaffolds and their predicted genes, with the exceptions of *FvVQ2*, *FvVQ22* and *FvVQ23* (Table 2). Interestingly, PCR amplifications of genomic DNA and cDNA with the specific designed primers showed that strawberry *VQ22* and *VQ23* failed to be detected in cDNA samples from both the diploid *F*. *vesca* cv. Reina de los Valles and *F*. ananassa cv. Camarosa in all tested tissues, developmental stages and treatment conditions (see further below). Using BLASTN, we also compared the curated *VQ* genes found by HMM3 on the Fv v2.0.a2 as well as the newest Fv v4.0.a1 genome annotation, with negative results for these two genes (Supplemental Table S2). Thus, we think that prediction of these two genes should be taken with caution.

**Table 2:** *Fragaria x ananassa VQ* genes and transcripts homologs to *FvVQs*. Asterisks indicate transcripts mapped on the FANhybrid r1.2 reference genome by BLAT, with alignments lengths of 95% and 90% identity. BLAT alignments can be found at https://www.rosaceae.org/analysis/230.

Name	*F*.*vesca homolog* gene	FANhybrid r1.2 scaffolds	FANhybrid gene id.	*Fragaria x ananassa* RefTrans V1	Domains
*FaVQ1*	*gene05795-v1*.*0-hybrid*	FANhyb_rscf00000599.1	*FANhyb_rscf00000599*.*1*.*g00006*.*1*	f.ananassa_gdr_reftransV1_0027312*	VQ
				f.ananassa_gdr_reftransV1_0053740*	VQ
*FaVQ2*	*gene12350-v1*.*0-hybrid*	FANhyb_icon00018020_a.1	*FANhyb_icon00018020_a*.*1*.*g00001*.*1*	no hits found	
*FaVQ3*	*gene17872-v1*.*0-hybrid*	FANhyb_icon00002146_a.1	*FANhyb_icon00002146_a*.*1*.*g00001*.*1*	f.ananassa_gdr_reftransV1_0042573	VQ
				f.ananassa_gdr_reftransV1_0062571	VQ
*FaVQ4*	*gene16338-v1*.*0-hybrid*	FANhyb_rscf00005480.1	*FANhyb_rscf00005480*.*1*.*g00001*.*1*	f.ananassa_gdr_reftransV1_0054430*	VQ
*FaVQ5*	*gene32666-v1*.*0-hybrid*	FANhyb_icon00017850_a.1	*FANhyb_icon00017850_a*.*1*.*g00001*.*1*	f.ananassa_gdr_reftransV1_0044247	VQ
*FaVQ6*	*gene02378-v2*.*0*.*a2-hybrid*	FANhyb_icon00041277_a.1	*FANhyb_icon00041277_a*.*1*.*g00001*.*1*	f.ananassa_gdr_reftransV1_0054841	VQ
		FANhyb_rscf00000298.1	*FANhyb_rscf00000298*.*1*.*g00005*.*1*	f.ananassa_gdr_reftransV1_0000040	VQ
*FaVQ7*	*gene19985-v1*.*0-hybrid*	FANhyb_rscf00000053.1	*FANhyb_rscf00000053*.*1*.*g00024*.*1*	f.ananassa_gdr_reftransV1_0018796*	VQ
*FaVQ8*	*gene02352-v2*.*0*.*a2-hybrid*			f.ananassa_gdr_reftransV1_0019517	VQ
*FaVQ9*	*gene17096-v2*.*0*.*a2-hybrid*	FANhyb_rscf00000298.1	*FANhyb_rscf00000298*.*1*.*g00005*.*1*	f.ananassa_gdr_reftransV1_0040065*	VQ
		FANhyb_rscf00000496.1	*FANhyb_rscf00000496*.*1*.*g00005*.*1*	f.ananassa_gdr_reftransV1_0059358	VQ
*FaVQ10*	*gene22762-v1*.*0-hybrid*	FANhyb_rscf00000136.1	*FANhyb_rscf00000136*.*1*.*g00007*.*1*	f.ananassa_gdr_reftransV1_0068457*	VQ
*FaVQ11*	*gene03724-v1*.*0-hybrid*			f.ananassa_gdr_reftransV1_0029239	VQ
*FaVQ12*	*gene25153-v1*.*0-hybrid*	FANhyb_rscf00000010.1	*FANhyb_rscf00000010*.*1*.*g00032*.*1*	f.ananassa_gdr_reftransV1_0019677*	VQ
*FaVQ13*	*gene26510-v2*.*0*.*a2-hybrid*	FANhyb_rscf00006122.1	*FANhyb_rscf00006122*.*1*.*g00001*.*1*	f.ananassa_gdr_reftransV1_0023329	VQ, LRR8, NB-ARC
				f.ananassa_gdr_reftransV1_0063702	VQ, LRR8, NB-ARC
				f.ananassa_gdr_reftransV1_0042571	VQ, NB-ARC
*FaVQ14*	*gene11973-v1*.*0-hybrid*	FANhyb_rscf00002726.1	*FANhyb_rscf00002726*.*1*.*g00001*.*1*	f.ananassa_gdr_reftransV1_0001655*	VQ
				f.ananassa_gdr_reftransV1_0058547*	VQ
*FaVQ15*	*gene22130-v1*.*0-hybrid*	FANhyb_rscf00001000.1	*FANhyb_rscf00001000*.*1*.*g00002*.*1*	f.ananassa_gdr_reftransV1_0059602	VQ
*FaVQ16*	*gene16601-v1*.*0-hybrid*	FANhyb_rscf00000324.1	*FANhyb_rscf00000324*.*1*.*g00002*.*1*	f.ananassa_gdr_reftransV1_0042218	VQ
*FaVQ17*	*gene00230-v1*.*0-hybrid*	FANhyb_icon00005101_a.1	*FANhyb_icon00005101_a*.*1*.*g00002*.*1*	f.ananassa_gdr_reftransV1_0073885	VQ
				f.ananassa_gdr_reftransV1_0013307	VQ
*FaVQ18*	*gene21583-v1*.*0-hybrid*	FANhyb_rscf00004997.1	*FANhyb_rscf00004997*.*1*.*g00001*.*1*	f.ananassa_gdr_reftransV1_0027413*	VQ
*FaVQ19*	*gene18133-v1*.*0-hybrid*			f.ananassa_gdr_reftransV1_0033646	VQ
*FaVQ20*	*gene22640-v1*.*0-hybrid*	FANhyb_icon00000069_a.1	*FANhyb_icon00000069_a*.*1*.*g00001*.*1*	f.ananassa_gdr_reftransV1_0038560*	VQ
*FaVQ21*	*gene15994-v1*.*0-hybrid*	FANhyb_icon00002699_a.1	*FANhyb_icon00002699_a*.*1*.*g00001*.*1*	f.ananassa_gdr_reftransV1_0069556	VQ
*FaVQ22*	*gene28736-v1*.*0-hybrid*			no hits found	
*FaVQ23*	*gene28758-v1*.*0-hybrid*			no hits found	
*FaVQ24*	*gene24219-v1*.*0-hybrid*	FANhyb_icon00016110_a.1	*FANhyb_icon00016110_a*.*1*.*g00001*.*1*	f.ananassa_gdr_reftransV1_0059293	VQ
*FaVQ25*	*gene14221-v1*.*0-hybrid*			f.ananassa_gdr_reftransV1_0013396	VQ

On the other hand, several homologous transcripts were found matching with the RefTrans V1 transcripts for many *FaVQs* indicating the existence of more alternative splicing forms in the strawberry octoploid than in the diploid species (Table 2), Thus, two different transcripts were found for genes *FaVQ1*, *FaVQ3*, *FaVQ6*, *FaVQ9*, *FaVQ14 and FaVQ17*, and three different transcripts for gene *FaVQ13*. It is worth noting that two out of the three different *FaVQ13* transcripts code for proteins preserving the full predicted domains structure while the third one lacks the LRR8 domain (see below). These findings evidence differences in the control of mRNA maturation of these genes between the two strawberry species at post-transcriptional level that remains to be further studied.

Curiously, Fv/FaVQ13 exhibited two domains found in the NBS-LRR class of R proteins: a NBS-ARC domain (PF00931) and a Leu-rich motif (LRR_8; PF13855). This unusual association of such domains with the VQ motif is novel but a similar architecture is also found in CDD/CDART in several *Oryza sativa* proteins corresponding to OsVQ34^36^, *Beta vulgaris* (XP_010673130.1) and *Chenopodium quinoa* (XP_021745766.1) (Supplemental Fig. S1). Moreover, in a preliminary screening of the Phytozome 12.1 database we have found two more proteins, from *M*. *domestica* (MDP0000284090) and *C*. *grandiflora* (Cagra.22718s0002.1) harbouring similar domains. Interestingly, R protein domains have also been reported in combination with WRKY domains within the R protein-WRKY class of WRKY TF^6,37^. By analogy, we propose the name of R protein-VQ for this novel class of VQ proteins. Besides, a variety of domains associated with the VQ were also found in proteins from different taxa. These findings reveal an unexpected structural complexity in the VQ protein family and suggest that their biological functions may be more diverse than we currently know.

### Phylogenetic and structural analysis of FvVQ proteins

**Figure 2:**
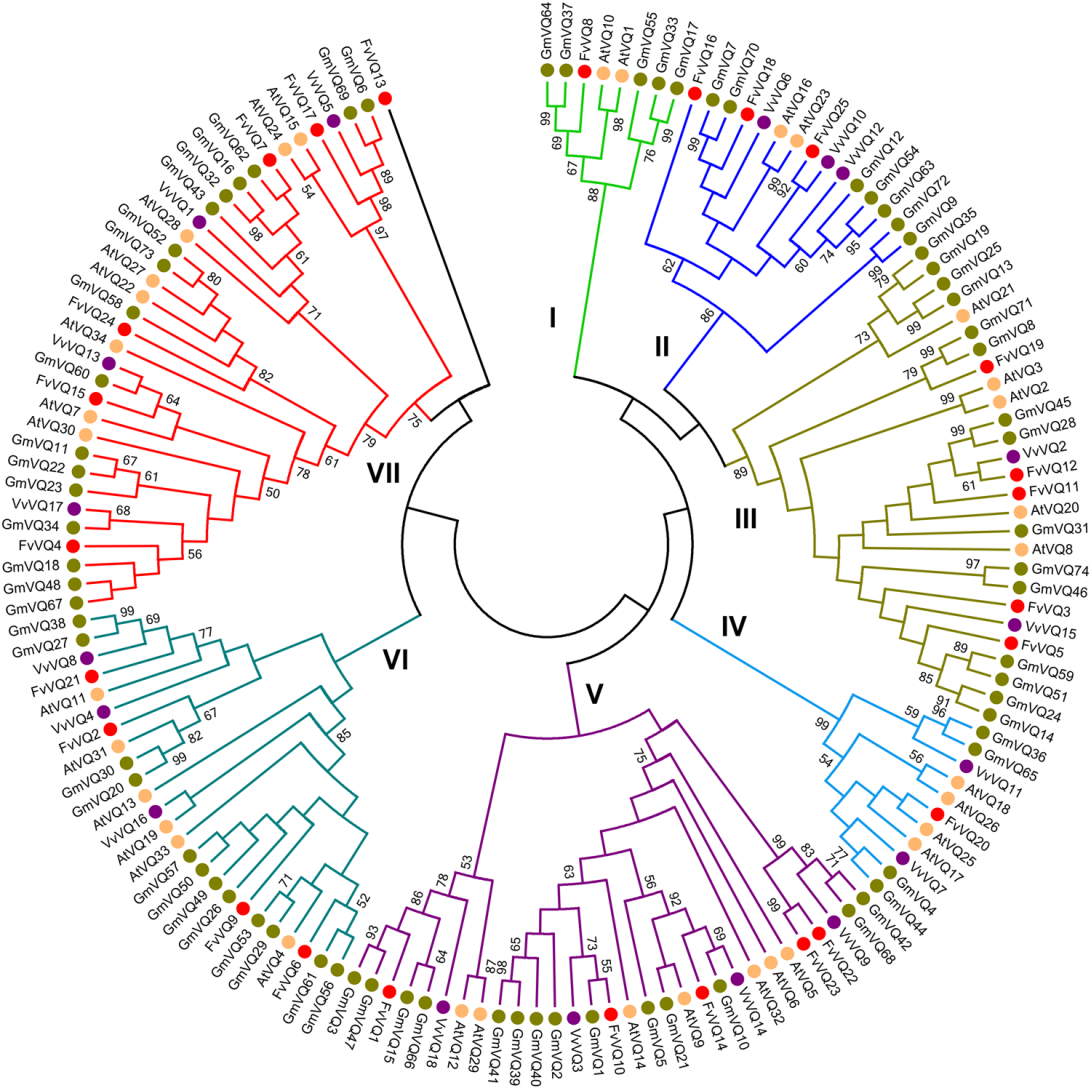
Phylogenetic analysis of strawberry, soybean, grapevine and *Arabidopsis* VQ proteins. The VQ domain of the four species was aligned by PROMALS3D and an unrooted tree was constructed using MEGA 7.014 by the neighbour-joining method (1000 bootstrap replicates). The groups obtained are sorted by colours and numbered in groups one to seven (I–VII).

**Figure 3:**
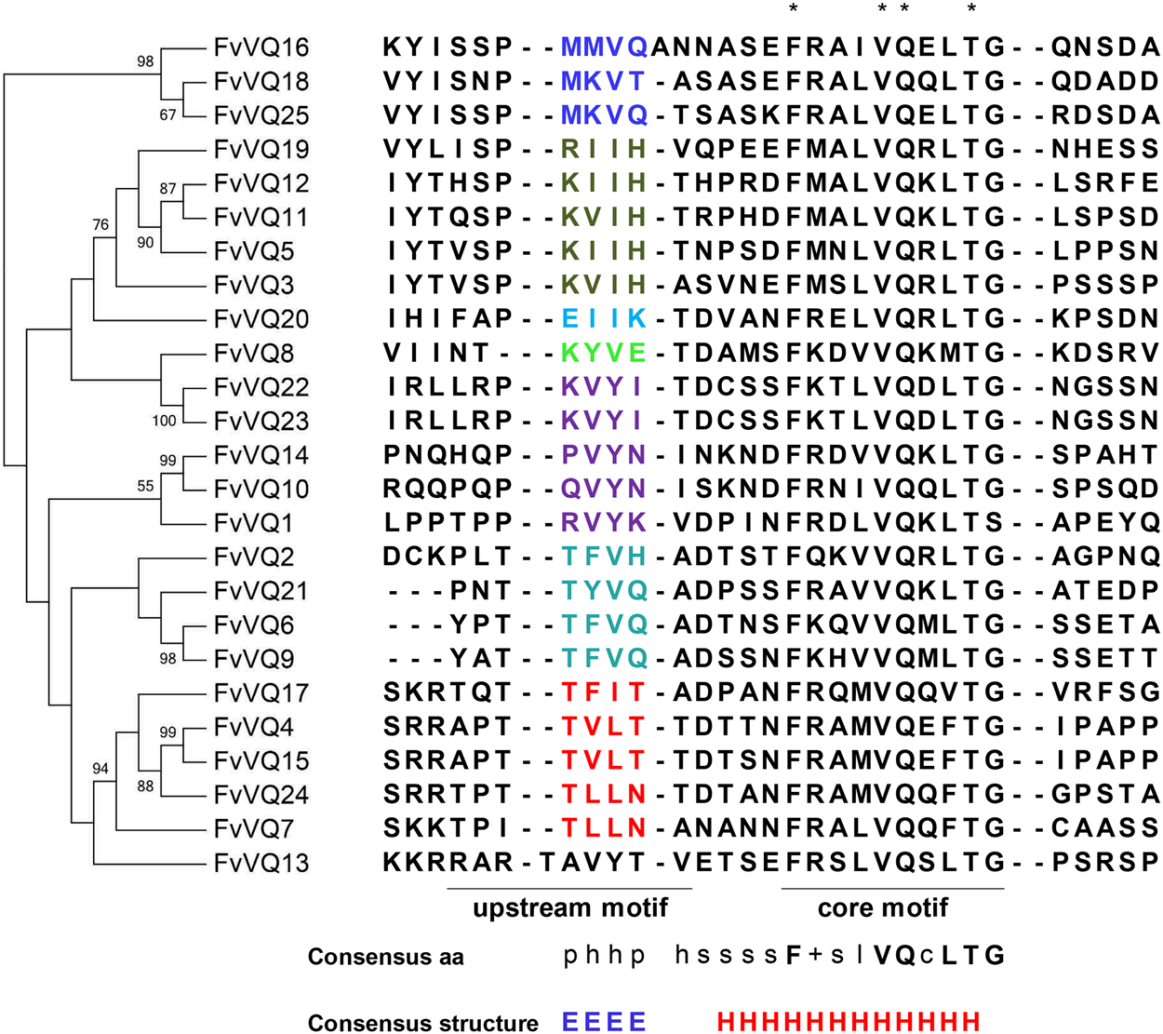
Phylogenetic analysis and multiple sequence alignment of the strawberry VQ protein domain. The VQ domain was aligned by PROMALS3D and an unrooted tree was constructed using MEGA 7.014 by the neighbour-joining method (1000 bootstrap replicates). The seven groups obtained are sorted by the same colours used in the four species tree. The upstream and core motifs of the aligned VQ domain are underlined. Consensus amino acids (aa) and secondary structure are shown. Highly conserved aa are noted by uppercase bold and asterisks. Consensus aa symbols (lowercase): p, polar residues (D,E,H,K,N,Q,R,S,T); h, hydrophobic residues (W,F,Y,M,L,I,V,A,C,T,H); s, small residues (A,G,C,S,V,N,D,T,P); +, positively charged residues (K, R, H); l, aliphatic residues (I,V,L); c, charged (D,E,K,R,H). Consensus structure symbols: E, β-sheet; H, α-helix.

In order to assess the evolutionary relationships of VQ proteins among species, previous studies have used either the full predicted VQ protein sequences^10,38–40^ or only the conserved amino acids of the aligned VQ domain^11,17,36,41^ to construct phylogenetic trees. In our study, we have taken into account all this information including the recently identified eight amino acids long upstream motif from the core VQ motif^10^, to perform a structure-based sequence alignment of the VQ domains from *F*. *vesca* in comparisons to *Arabidopsis*, soybean and grapevine by PROMALS3D. These results were subsequently used to construct phylogenetic trees by the N-J method in MEGA, either comparing the four mentioned species (Figs 2 and S2), or for the strawberry FvVQ alone (Fig. 3). In both cases, the results show a distribution of the VQ proteins into seven groups, with a group-specific arrangement of the upstream motif sequences (Supplemental Fig. 2 and Fig. 3). FvVQ13 is classified alone as outgroup because of its sequence divergence, but with low bootstrap support. A phylogenetic tree was also constructed using the full protein sequences of the FvVQs and a similar clustering in seven clades was obtained preserving the group-specific arrangement of the VQ motif sequences (Supplemental Fig. S3), supporting the observation that the VQ domain is the most important determinant for the phylogenetic relationships in the VQ protein family also in strawberry^10^.

The structurally-driven sequence alignment reveals the β-sheet-loop-α-helix consensus structure for the VQ domain described by Zhou *et al*.^10^, who also suggested a critical role of the upstream motif in the WRKY-VQ interaction by modulating the core motif binding specificity, is also preserved in the four species here compared. It is worth it to mention that this group distribution does not reflect any kind of intra-group uniformity in terms of exclusivity of binding capabilities to WRKY nor transcriptional regulation activity (activation or repression), at least for those VQs reported in *Arabidopsis*. For example, several VQs placed in different groups interacted with the C-terminal domain of WRKY25 or WRKY33 in YTH^8^, while VQs grouped together can exhibit activation, repression or no transcriptional regulation activities in LUC transient expression assays^15^. This suggest that the VQ motif itself is not the only determinant for the VQ protein functionality, as well as the WRKY binding specificity may be shared by different VQ groups with small differences in their upstream motif sequences.

Besides, complementary structural analysis were carried out in the RaptorX Structure Prediction web server^42^ using the full VQ protein strawberry and *Arabidopsis* sequences. The results confirmed the consensus secondary structure along the VQ domain, with the notable exception of the upstream motif of FvVQ13, where the β-sheet structure was not predicted. Also, these results, revealed that most of the FvVQ and AtVQ proteins share long protein tracks structurally disordered. Intrinsically disordered proteins (IDPs) are abundant and important components of cellular signalling pathways combining, among others characteristics, the presence of specific recognition motifs, accessible sites for post-translational modifications and a high degree of structural flexibility that allows the interaction with more than one different target^43^. Most of the biological processes enriched in IDPs found in plants are related to environmental stimuli perception and stress responses, involving protein families like dehydrins, GRAS, NAC and bZIP^44^. Consequently, IDP regions could provide new VQ-protein interaction properties to FvVQ and AtVQ proteins making them predictable components of cellular signalling pathways, which can bind different proteins other than WRKY TFs. Thus, MPK3/6 and MPK4 can interact with and phosphorylate respectively, the *Arabidopsis* MVQ subgroup^21^ and MSK1 (AtVQ21)^20^. Also, AtVQ29 has been shown to bind PIF1^15^ and AtVQ32 can bind NDL1^8^. Moreover, *Arabidopsis* VQ12 and VQ29 can bind with themselves or each other to form homo- or heterodimers, as well as with other VQs, being the C-terminal regions responsible for the VQ-VQ interaction^18^. The exact domains implicated in these protein-protein associations, if any, remain to be further identified.

Additional motifs included in the FvVQ predicted proteins were analysed by the MEME suite (Supplemental Fig. S3 and Supplemental Table S3). The conserved VQ domain, containing both the upstream and the core motifs, was clearly recognized in all of the clades, as well as additional motifs featured in the different proteins. Most of such them were also found in the VQ sequences from other species by BLASP searches. Although these motifs potentially indicate function diversity among the VQ proteins, no specific roles are evident or have been reported to date.

### Identification of strawberry FvVQ homologs in *Arabidopsis* VQ proteins

In order to infer potential functions of the strawberry VQ proteins, the homology between AtVQs and FvVQs was first investigated using the best reciprocal BLAST hit (BRH) method (Supplemental Table S4), complemented with the OrthoMCL database and the phylogenetic trees here generated. The result is summarized in Table 3. A consensus among the three methods employed was obtained except for FvVQ5, FvVQ7, FvVQ9 and FvVQ15, either due to the lack of a conclusive best reciprocal AtVQ partner or because the best BLASTP hits did not belong to the same OrthoMCL groups. However, we have assigned putative AtVQ homologs to them taking into account the following considerations. FvVQ3 and AtVQ21 shared the best reciprocal values (bit scores of 65.1 for FvVQ3 as query, and 89.4 for AtVQ21 as query) and FvVQ5 also showed the highest sequence homology values with AtVQ21 but the second best for the reciprocal (bit scores of 83.2 for FvVQ5 as query, and 70.5 for AtVQ21 as query). However, these three proteins clustered together (Fig. 2). Therefore, AtVQ21 was also assigned to FvVQ5. In a similar way, AtVQ4 was assigned to FvVQ6 and FvVQ9 (respectively, bit scores of 152 for FvVQ6 as query and 206 for AtVQ4 as query, and of 134 for FvVQ9 as query and 171 for AtVQ4 as query), which also are clustering together. Also, FvVQ7 and FvVQ15 are clustered together within the same clade. However, AtVQ7 was assigned to FvVQ15 as they shared the best reciprocal hits (bit scores of 91.3 for FvVQ15 as query, and 90.9 for AtVQ7 as query). On the contrary, we tentatively assigned AtVQ30 to FvVQ7 only on the basis of the best (non-reciprocal) BLASTP matching pairs (bit scores of 47.4 for FvVQ7 as query) as the best reciprocal hit for AtVQ30 was FvVQ15 (bit scores of 61.6 for AtVQ30 as query), which had already AtVQ7 assigned as the best reciprocal hit option. On the other hand, FvVQ8 and FvVQ16 were classified within group OG5_244916, with no corresponding members of the AtVQ family. We propose AtVQ10 for FvVQ8 as they shared the best reciprocal hits (bit scores of 50.8 for FvVQ8 query, and 51.2 for AtVQ10 as query). Also, FvVQ16 matched the best (non-reciprocal) with AtVQ23 (bit scores of 48.5 for FvVQ16 as query) but AtVQ23 did it both with FvVQ18 as the best hit and FvVQ16 as the best third (bit scores of 55.5 and of 41.2, respectively for AtVQ23 as query). As long as FvVQ16 and FvVQ18 were clustered together and FvVQ18 had already AtVQ16 assigned as its best reciprocal hit (bit scores of 59.3 for FvVQ18 query, and 62.8 for AtVQ16 query), AtVQ23 was assigned to FvVQ16. For the remaining FvVQs, the OrthoMCL grouping, BRH results and the phylogenetic clustering obtained were coincident. For FvVQ1 two putative orthologs were found, AtVQ12 and AtVQ29, two closely related proteins, but showing the highest homology to AtVQ12. On the other hand, FvVQ13 was classified as member of the group OG5_134032, together with several R-proteins as the RPP13-like protein 1 (At3g14470.1), which was the best BLASTP hit found in the *Arabidopsis* proteome. Little homology of FvVQ13 with other VQ members was found but restricted to the VQ domain context.

**Table 3:** Orthologs between strawberry and Arabidopsis VQ proteins. The best reciprocal BLASTP hits are marked by asterisks.

OrthoMCL group	AtVQ members	FvVQ protein	Arabidopsis ortholog
OG5_177680	AtVQ12, 29	FvVQ1	AtVQ12*, AtVQ29
OG5_213230	AtVQ31 (MVQ6)	FvVQ2	AtVQ31*
OG5_213152	AtVQ21 (MKS1)	FvVQ3	AtVQ21*
OG5_212399	AtVQ20	FvVQ12	AtVQ20*
OG5_134032	no AtVQs in this group	FvVQ13	At3g14470.1
OG5_190867	AtVQ9 (MVQ10)	FvVQ14	AtVQ9*
OG5_164495	AtVQ15,24	FvVQ17	AtVQ24*
OG5_177741	AtVQ17,25	FvVQ20	AtVQ25*
OG5_147155	AtVQ2,3	FvVQ5	AtVQ21
OG5_170456	AtVQ14 (IKU1, MVQ9)	FvVQ10	AtVQ14*
NO_GROUP	AtVQ5,13,19,28,30,33	FvVQ9	AtVQ4
OG5_178155	AtVQ34	FvVQ4	AtVQ34
		FvVQ15	AtVQ7*
OG5_150231	AtVQ4 (MVQ1), 11 (MVQ5)	FvVQ6	AtVQ4*
		FvVQ21	AtVQ11*
OG5_170861	AtVQ22 (JAV1), 27	FvVQ7	AtVQ30
		FvVQ24	AtVQ22*
OG5_244916	no AtVQs in this group	FvVQ8	AtVQ10*
		FvVQ16	AtVQ23
OG5_212106	AtVQ8 (PDE337)	FvVQ11	AtVQ8*
		FvVQ19	AtVQ8
OG5_189669	AtVQ16 (SIB2), 23 (SIB1)	FvVQ18	AtVQ16*
		FvVQ25	AtVQ23*

### Predicted *Cis*-control elements within the regulatory region of *FvVQ* genes

To gain insight into the regulation of the strawberry *VQs* gene expression, the putative regulatory regions of *FvVQ* genes were investigated searching for known *cis*-elements. Thus, the 1500 bp upstream regions preceding all *FvVQ* coding sequences (except 842 bp for *FvVQ10*, 502 bp for *FvVQ*17 and 1185 bp for *FvVQ*23) were screened in the PLANTCARE database. A wide range of biotic and abiotic stresses, elicitor, phytohormone responsive elements and developmental regulatory sequences were predicted (Supplemental Table S5). Interestingly, there are abundance of light, abscisic acid (ABRE), gibberellins (GARE and P-box) and auxin (TGA) responsive elements, which have already been described as important factors in the development and ripening of fruit receptacle and achenes in this non-climacteric fruit^45–47^. Also remarkable is the presence in most of the promoters here analysed, of two elements involved in endosperm gene expression, GCN4- and Skin-1 motifs, suggesting that a number of *FvVQ* genes could be expressed in the endosperm of achenes.

On the other hand, known response elements to salicylic acid (TCA-element) and methyl jasmonate (CGTCA- and TGACG-motifs) were also found in most of the *FvVQs*, suggesting a potential role of these genes in strawberry plant defence. Furthermore, several binding site sequences for important plant transcription factors (TFBS) including NAC, bZIP, bHLH, MYB and WRKY, were found within many *FvVQ* promoter regions (Supplemental Table S6). Given that WRKY TFs can bind to alternative sequences other than the consensus TTGACY (where Y = C/T), we also searched for WK-boxes (TTTTCCAC), recognized by WRKY TFs carrying the WRKYGKK motif^48^ and WT-boxes (YGACTTTT), recognized by the *Arabidopsis* WRKY70^49^. Results are summarized in Supplemental Table S7. One or more W-boxes were found in 23 out of 25 *FvVQs* promoters, with exceptions of *FvVQ21* and *FvVQ25*. No WK-boxes were found, but single WT-box sequences are present in *FvVQ2*, *FvVQ3*, *FvVQ5*, *FvVQ12* and *FvVQ24*. The theoretically-expected frequencies for the consensus W-box and WT-box for both DNA chains within the 1.5-kb promoter sequences of *FvVQs* (GC%: 38.9793 for the FraVesHawaii_1.0 assembly) are roughly 1 per 1.7-kb and 17.4-kb, respectively. Based on this, we consider that the presence of two, or more W-box sequences as well as single WT-boxes within the 1500 bp upstream region of *FvVQ* genes is remarkable and suggests that the expression of some strawberry *VQs* may be regulated by WRKY TFs, including strawberry WRKY70-like TFs.

### Expression profiles of *FaVQ* genes in different tissues and fruit ripening stages

**Figure 4:**
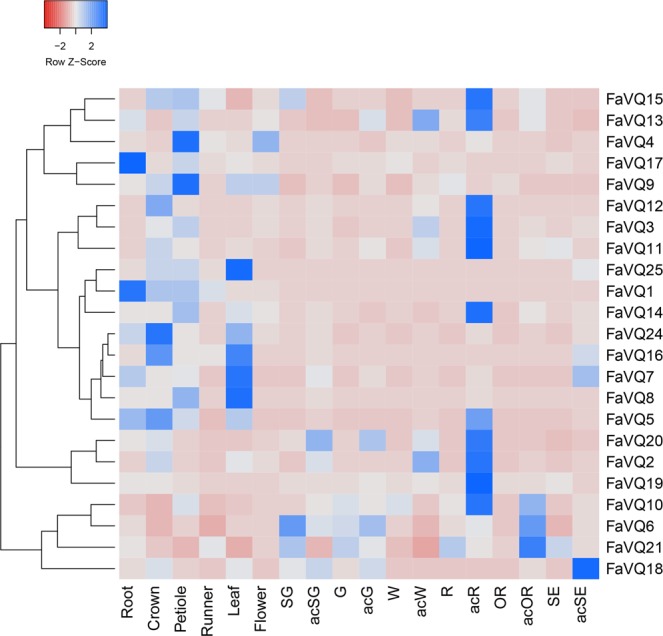
Heatmap representation of the *FaVQs* expression profiles in different tissues and fruit ripening stages. Abbreviations (fruit receptacles): **SG**, small green; **G**, green; **W**, white; **R**, red (ripe); **OR**, overripe; **SE**, senescent; achenes from the corresponding stages are preceded by an **ac**- prefix.

The expression profiles of *VQ*s were analysed in the octoploid strawberry species (cv. Camarosa) by RT-qPCR in different tissues, as well as fruit receptacles and achenes along several ripening stages (Fig. 4). Results indicate that *FaVQ1*, *FaVQ4*, *FaVQ8*, *FaVQ9*, *FaVQ16*, *FaVQ17*, *FaVQ24 and FaVQ25* are preferentially expressed in roots, crown, petioles and leaves. Thus, *FaVQ17* and *FaVQ1* were the most expressed genes in root, *FaVQ24* and *FaVQ16* in crown, and *FaVQ7*, *FaVQ8*, *FaVQ16* and *FaVQ25*, in leaf, *and FaVQ4* and *FaVQ9* both in petiole and flowers, being these two later genes the only ones notably expressed in flowers. Genes *FaVQ7* and *FaVQ16* were not preferentially expressed in fruit but a higher expression in senescent achene. A different set of genes, including *FaVQ2*, *FaVQ3*, *FaVQ10*, *FaVQ11*, *FaVQ12*, *FaVQ13*, *FaVQ14*, *FaVQ15*, *FaVQ19*, and *FaVQ20*, showed preferential expression in fruit, particularly in red ripe achenes. Only the expression of genes *FaVQ6*, *FaVQ10* and *FaVQ21*, was preferentially detected in fruit receptacle and achene, being *FaVQ6* highly expressed in receptacle of small green fruit, *FaVQ10* in red achene and *FaVQ21* highly expressed in over-ripe achene. It is worth to notice that *FaVQ18* was the highest expressed in senescent achene. It is tentative to propose that the changes observed in the expression pattern of these *FaVQs* during the fruit ripening stages should be associated to changes in the level of hormones along such a fruit process already described. Thus, it has been described that an initial strong increase of auxin occurs, while gibbellerins declines progressively during the ripening process, at the time that abscisic acid (ABA) increases coinciding with the colour development phase^46^. In addition, auxin and ABA are found at higher levels in achenes than in receptacles, depending of the developmental stage. Further studies are needed to provide a better understanding of putative roles of FaVQs in this processes.

### Expression analysis of *FaVQ* genes in fruit in response to *C*. *acutatum* infection

**Figure 5:**
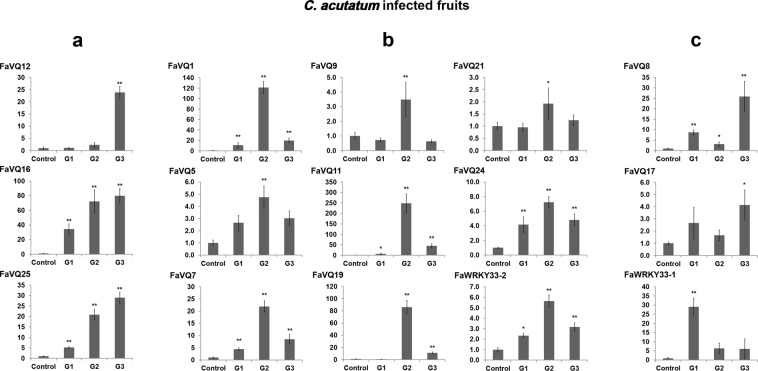
Expression profiles of *FaVQ* genes in anthracnose diseased fruits. The panels (a–c) show the different expression patterns described in the main text. Only the genes whose expression were significantly different from the control at any experimental point (Dunnett’s test) are shown. Mean, standard error (SE) and significant differences of *P ≤ 0.05 and **P ≤ 0.01 are represented.

To explore putative implications of FaVQs in the strawberry defence response against *C*. *acutatum*, the expression of *FaVQ*s was analysed in fruit of *F*. *ananassa* cv. Camarosa showing increasing symptoms of anthracnose disease. Since *WRKY33* has been described as a key component in plant defence response to necrotrophic fungi in other plants^50,51^ and its interaction with several VQ proteins has been well established^8,13,21^, the expression of *FaWRKY33-1* and *FaWRKY33-2*, two known strawberry *WRKY33* orthologs^33^ was also studied. Our results showed that the expression of 14 out of the 25 *FaVQs* as well as the two *FaWRKY33s*, was clearly up-regulated in strawberry during pathogen infection but different expression patterns were detected (Fig. 5). Thus, a continuous increase of gene expression, which correlates with the infection-induced tissue damage (stages G1 to G3), was found for genes *FaVQ12*, *FaVQ16* and *FaVQ25* (Fig. 5a). A second group of genes including *FaVQ1*, *FaVQ5*, *FaVQ7*, *FaVQ9*, *FaVQ11*, *FaVQ19*, *FaVQ21*, *FaVQ24* and *FaWRKY33*.2 showed a maximum accumulation of transcripts at G2 stage but their expressions were reduced at later stages (Fig. 5b). On the other hand, the expression pattern of *FaVQ8* and *FavQ17* was very similar and transcripts slightly accumulated at G1 stage, decreasing gene expression at G2 stage before increasing again to a top level at G3 stage (Fig. 5c). Contrastingly, *FaWRKY33*.*1* showed the highest expression at G1 stage and reduced its expression at G2 and G3 stages. The topmost relative expression level (ranging from 90 to 250 times higher than the control uninfected) was for genes *FaVQ1*, *FaVQ11*, *FaVQ16* and *FaVQ19*, compared to genes *FaVQ2*, *FaVQ8*, *FaVQ12*, and *FaVQ25* (ranging from 20 to 25 times higher than the control uninfected) or genes *FaVQ5*, *FaVQ9*, *FaVQ17*, *FaVQ21*, and *FaVQ24* (ranging from 1.6 to 7 times higher than the control uninfected). Transcription of genes *FaVQ2*, *FaVQ3*, *FaVQ4*, *FaVQ6*, *FaV10*, *FaVQ13*, *FaVQ14*, *FaVQ15*, *FaV18*, *FaVQ20*, remained unchanged, while *FaVQ22* and *FaVQ23* were not detected.

Our results also show that a wide group of *FaVQs* and the two *FaWRKY33* genes were up-regulated after *C*. *acutatum* infection, and suggest putative implications of these genes in the strawberry defence response. It is well known that AtWRKY33 works as a positive regulator of defence response in *Arabidopsis*, which can interact with partners controlling its activity. Thus, MKS1 (AtVQ21) and SIB1 (AtVQ23) and SIB2 (AtVQ16) are known regulators of AtWRKY33 activity and *SIB1*, *SIB2 and AtWRKY33* showed similar expression patterns and were markedly induced after *B*. *cinerea* infection^16^. Accordingly, *FaVQ5* (*MKS1* ortholog), *FaVQ16* and *FaVQ25 (SIB1* orthologs) and the two *FaWRKY33s* were highly up-regulated in strawberry infected fruit while *FaVQ18* (*SIB2 ortholog*) expression did not change significantly.

Recently, a new set of *Arabidopsis* VQs, have been found to interact with different WRKYs, forming a variety of complexes and being the substrate of MAPKs^21^. Such as protein complexes are depending on spatio-temporal *VQP* and *WRKY* expression patterns and defence gene transcription can be modulated by changing the composition of the complexes^21^. Thus, VQ8 (MSK1 homolog) was found to interact with WRKY33 as well as with MPK4, suggesting that it may have similar functions to MKS1 (AtVQ21) in defence responses. Curiously, the strawberry *VQ8* orthologous genes, *FaVQ11* and *FaVQ19*, were highly induced in fruit after *C*. *acutatum* infection (Fig. 5). It is worth to note that AtVQ8 and the strawberry FaVQ11 and FaVQ19 share high homology with MKS1 (AtVQ21) and FaVQ3 and FaVQ5, respectively, and all of them belong to the same clade/cluster (Figs 2 and 3). Another strawberry WRKY33-interacting VQ orthologous gene, *FaVQ7* (*AtVQ30* ortholog), was also detected to be up-regulated.

The expression of some non WRKY33-interacting VQs was also up-regulated in strawberry in response to *C*. *acutatum* infection. Thus, the expression of *FaVQ12*, the *VQ20* ortholog acting as negative regulator of the defence responses^8^, and *FaVQ17* (*VQ24* ortholog) were drastically increased in G3 infected fruit (Fig. 5a,c). Also *FaVQ24*, the JAV1 ortholog acting as negative controller of the JA-mediated plant defence response by interacting with WRKY51^52,53^, was significantly up-regulated in strawberry (Fig. 5b). In addition, *FaVQ1* was also strongly induced in fruit upon *C*. *acutatum* infection, in agreement with the results described in Arabidopsis^18^ and rice^36^ for its *VQ12* and *VQ29* orthologs, which negatively regulate the defence response against *B*. *cinerea* in a partially dependent JA-signalling pathway manner. Because *FaVQ1* is also homolog to *VQ29*, which bind WRKY33 in YTH assays^8^, interaction with strawberry FaWRKY33 proteins may not be discarded. On the other hand, *AtVQ10* (*FaVQ8* ortholog), has been recently described to enhance tolerance to oxidative stress^54^, and to participate in the defence response to pathogens^55^. Thus, At*VQ10* has been shown to interact with *WRKY8* to modulate basal defence against *B*. *cinerea*^55^. Curiously, in our study, the strawberry FaVQ8 ortholog was up-regulated after *C*. *acutatum* infection. In addition, other strawberry WRKYs-interacting VQs orthologous genes like *FaVQ9* (*MVQ1* ortholog) and *FaVQ21* (*MVQ5* ortholog) were also up-regulated. Contrastingly, the expression of some other strawberry WRKY-interacting VQ orthologs, was not altered after pathogen infection. Thus, *FaVQ2* (*MVQ6* ortholog), *FaVQ14* (*MVQ10* ortholog), *FaVQ20* (VQ25 ortholog, which negatively regulates defence response against *P*. *syringae* but not against *B*. *cinerea*^8^), *FaVQ15* (*VQ7* ortholog, of unknown function) and *FaVQ3* (another *MKS1* ortholog) did not change their expression in response to *C*. *acutatum*.

Strawberry *FaVQ* orthologous of *VQ* genes with unknown functions in defence response to date, like *FaVQ4* (*VQ34* ortholog), *FaVQ10* (*IKU1* ortholog) and *FaVQ13* (R protein-VQ) did not change their expression pattern in response to *C*. *acutatum* infection. Interestingly, the rice gene *OsVQ34*, coding for a FaVQ13 structurally-related protein, was no induced in response to both compatible and incompatible strains of *X*. *oryzae* (Xoo) suggesting that R protein-VQs may not be regulated in response to pathogens at the transcriptional level^36^.

### Expression analysis of *FaVQ* genes in response to SA and MeJA treatments

**Figure 6:**
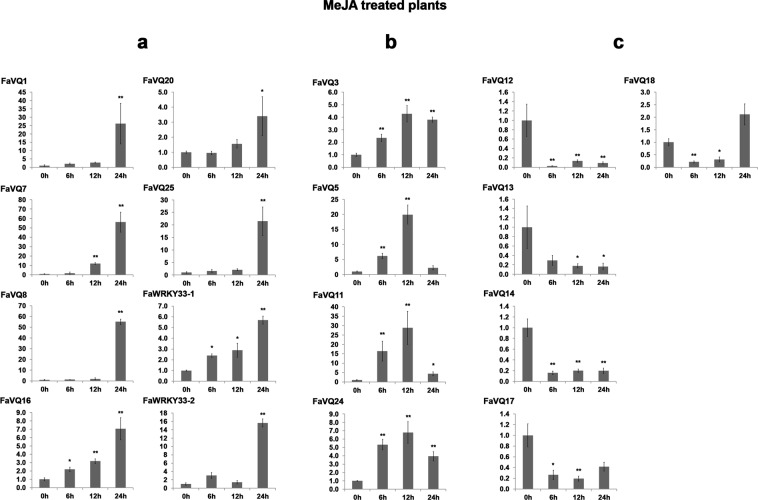
Expression profiles of *FaVQ* genes in MeJA (2 mM) sprayed *in vitro* plants. The panels (a, b, c) show the different expression patterns described in the main text. Only the genes whose expression were significantly different from the control at any experimental point (Dunnett’s test) are shown. Mean, standard error (SE) and significant differences of *P ≤ 0.05 and **P ≤ 0.01 are represented.

The responsiveness of the *FaVQ* and the two *FaWRKY33* genes to the exogenous application of SA and MeJA (the biologically active derivative of jasmonic acid), the two main activators of central defence signalling pathways which regulate responses to pathogens in many plants, was also studied by gene expression analyses. In response to MeJA, the expression of 9 out of the 25 *FaVQs* and of both *FaWRKY33* genes was up-regulated, and 6 other *FaVQ* genes were down-regulated (Fig. 6). Thus, the expression pattern of *FaVQ1*, *FaVQ7*, *FaVQ8*, *FaVQ16*, *FaVQ20*, *FaVQ25* and *FaWRKY33*.*2* was similar and a very high increase of transcripts was observed only at 24 h after treatment (Fig. 6a). A similar expression pattern was also found for genes *FaVQ16* and *FaWRKY33*.*1* and a continuous and significant increase in gene expression was observed from 6 h to 24 h after treatment. In addition, transcripts of genes *FaVQ3*, *FaVQ5*, *FaVQ11* and *FaVQ24* started to accumulate at 6 h after MeJA treatment but their highest expression levels were observed at 12 h, diminishing later to lower levels (Fig. 6b). Contrastingly, the expression of a third group of genes including *FaVQ12*, *FaVQ13*, *FaVQ14* and *FaVQ17* was strongly down-regulated at any times after MeJA treatment (Fig. 6c).

**Figure 7:**
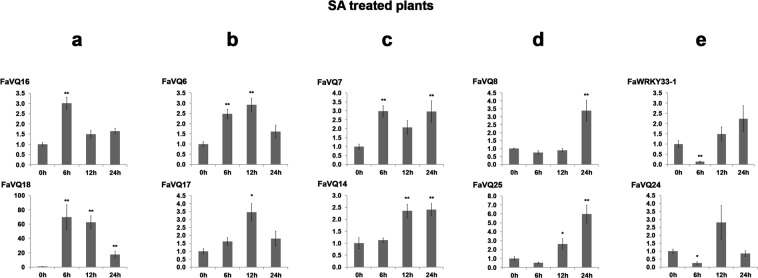
Expression profiles of *FaVQ* genes in SA (5 mM) sprayed *in vitro* plants. The panels (a–e) show the different expression patterns described in the main text. Only the genes whose expression were significantly different from the control at any experimental point (Dunnett’s test) are shown. Mean, standard error (SE) and significant differences of *P ≤ 0.05 and **P ≤ 0.01 are represented.

Responsiveness to SA was only detected for 9 out of the 25 *FaVQ* genes tested and the *FaWRKY33*-1 gene. Thus, the expression of *FaVQ16* and *FaVQ18* was significantly and quickly induced to the highest level at 6 h after SA treatment but continuously diminished after that, at later times (Fig. 7a). A similar pattern was detected for genes *FaVQ6* and *FaVQ17*, and a very significant increase was only detected at 12 hours (Fig. 7b). A much lower but significant up-regulation was also detected at any time for *FaVQ7* and *FaVQ14* (Fig. 7c) and significant increase was only detected at 24 h after SA treatment for genes *FaVQ8* and *FaVQ25* (Fig. 7d). A significant decrease of gene expression was detected at 6 h for *FaVQ24* and *FaWRKY33*-1 (Fig. 7e). No changes in the expression was detected for other *FaVQs* after SA treatment.

Contrastingly, the *FaVQ18* expression (*AtVQ16/SIB2* ortholog) was early down-regulated by MeJA but highly induced by SA treatment in strawberry, while *SIB1* orthologs *FaVQ16* and *FaVQ25* were up-regulated by both phytohormones. In addition, *FaVQ7* and *FaVQ8* were highly up-regulated by MeJA but only slightly induced by SA. Accordingly to the results reported in *Arabidopsis* for *JAV1*^52^, its strawberry *FaVQ24* ortholog was early up-regulated by MeJA. However, SA treatment induced a transient but significant down regulation at 6 h. Also, coinciding with the results obtained for *VQ12* in *Arabidopsis*^18^, the expression of strawberry *FaVQ1* was up-regulated by MeJA. However, *FaVQ12*, the strawberry ortholog of *VQ20* (another known negative regulator of defence^8^), was down-regulated in response to MeJA and not altered by SA treatment. Intriguingly, *FaVQ13* (LRR8-NBS-ARC-VQ protein) expression was highly down-regulated by MeJA treatment, while not responsiveness to SA treatment was detected. On the other hand, *FaVQ6* and *FaVQ14* were down-regulated by MeJA but significant up-regulation to low levels by SA treatment was detected.

These results are summarized in Fig. S4, grouping the *FaVQ* up-regulated genes by the different treatments, as well as the enriched regulatory *cis*-elements found by homology with their *FvVQ* orthologs.

### Network interaction analysis of the FaVQ proteins and FaWRKY33 in the response of strawberry to anthracnose disease

**Figure 8:**
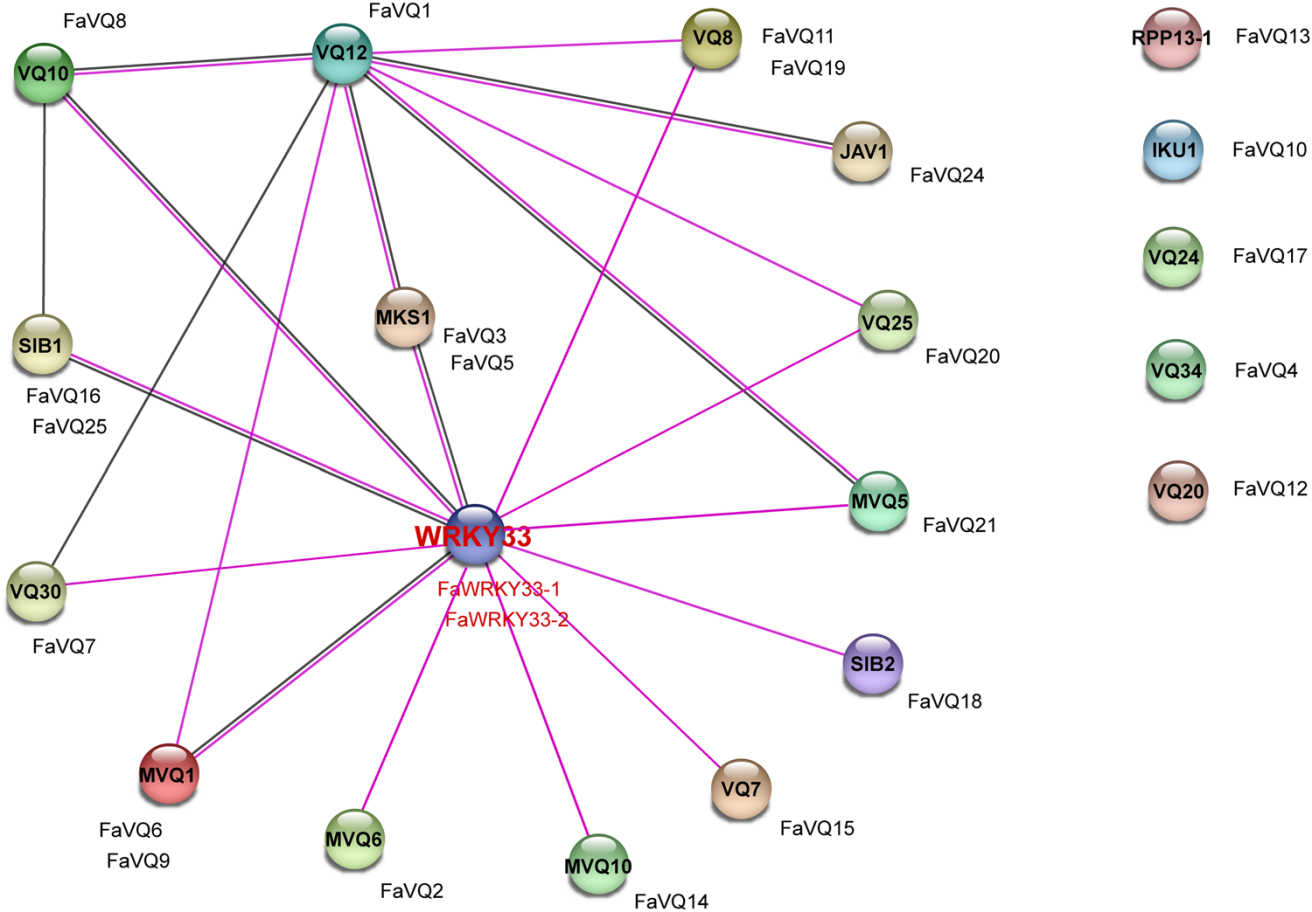
Functional interaction network of FaVQ proteins and FaWRKY33 based in their *Arabidopsis* orthologs. Nodes are connected by lines indicating experimentally determined interactions (purple) or co-expression (black). Disconnected FaVQ proteins at right side doesn’t have known relationships with WRKY33. The original figure was constructed in STRING 10.5 using medium confidence level (0.400) and experiments and co-expression as active interaction sources, then corrected to depict all the known experimental VQ interactions published to date.

To better understand the complex relationships that the strawberry VQ proteins can establish, we constructed a functional protein association network using STRING 10.5, based on the known interactions of the *Arabidopsis* orthologs uncovered by previous works^8,13,18,21^ and centred in their interactions with WRKY33 TFs. The Fig. 8 shows the intricate interactions among FaVQs as positive or negative regulators of the transcriptional activity of FaWRKY33s. A striking fact is that, while FaVQ1 (VQ12 ortholog) seems not able to bind directly to FaWRKY33s, represent a key node for other VQ members that at the same time interact with FaWRKY33s. Accordingly, it has been previously reported that VQ12 have ability to bind to other WRKY33-interacting VQs like MKS1 and its homologs VQ8, VQ10, VQ25 and VQ30, as well as to MPK3/6-targeted VQPs (MVQ1 and MVQ5)^18,21^. Notably, VQ12 also interact with JAV1, another JA-responsive negative regulator of defence against pathogens. Altogether, it can be speculated that FaVQ1 (VQ12 ortholog) may operate as an important node for a fine regulation of the JA-mediated response in strawberry by affecting the WRKY33-interacting VQs network. Besides, FaVQ1 could interact specifically with other members of the WRKY TF family, and/or non WRKY33-interacting VQ proteins as JAV1, and have additional roles in regulating gene expression of specific JA-responsive genes involved in the defence against many pathogens. The mechanisms of such regulation, that may imply VQ-VQ protein interactions, would add more complexity to the known models of VQ, WRKY and MPK protein interactions proposed previously^22^.

## Conclusions

A total of 23 VQ encoding genes were confirmed in the genomes of both the wild and the cultivated strawberry using the latest genome annotations and RNAseq. One of the strawberry VQs, Fv/FaVQ13, showed an unusual association with NBS-ARC and LRR8 domains. This new class is named here as R protein-VQ, by analogy with the R protein-WRKY, previously discovered. Also, other VQ proteins with this particular structure have been identified within other species proteomes. None of these proteins have been functionally characterized to date and we only can speculate about their possible roles, but the presence of NBS and LRR domains is expected to be related with pathogen recognition and the regulation of subsequent defence responses.

Strawberry orthologs to main *Arabidopsis* defence-related *VQ*s were found (*MKS1*, *SIB1*, *SIB2*, *JAV1* and *VQ12*, among others), indicating that analogous regulatory mechanisms of defence may exist in these two species. The expression profiles of the *FaVQs* showed tissue- and fruit ripening-dependent patterns. In addition, most of them were regulated in response to anthracnose, as well as to SA and MeJA hormonal treatment, suggesting a role in the strawberry defence responses. These results, lead to consider *FaVQs* as valuable target genes for further functional studies to address breeding programs to improve resistance in this crop.

## Materials and Methods

### Plant material and treatments

Field-grown strawberry plants (*Fragaria* x ananassa Duch cv. Camarosa) tissues and fruits were collected for gene expression analysis: roots, crown, petiole, leaf, mature flowers and fruits (receptacles and achenes) in different developmental stages. Naturally infected red strawberry fruits (cv. Camarosa) showing symptoms of anthracnose were collected and pooled into four groups representing increasing stages of disease development as described^56^: control (healthy) and grades 1 to 3 of infection (G1 to G3, corresponding to ID1 to ID3 in the referred paper). For phytohormone treatments, four weeks old *in vitro* Camarosa plants, growing in solid N30K medium^57^, were sprayed with mock, 5 mM salicylic acid (SA) or 2 mM methyl jasmonate (MeJA). Replicates were harvested at 6, 12 and 24 hours. All samples were frozen in liquid nitrogen and stored at −80 °C until RNA purification.

### Identification and molecular characterization of the VQ Family in *F*. *vesca* and *F*. *ananassa*

The previously published *Arabidopsis thaliana*, soybean and grapevine sequences of VQ proteins used in this study were downloaded from their original sources^8,10,11^. *Fragaria vesca* (genome v2.0.a2 and v4.0.a1) and *Fragaria* x ananassa (FANhybrid_r1.2 and GDR *Fragaria x ananassa* RefTrans V1) sequences are available online in the Genome Database for Rosaceae website (https://www.rosaceae.org/). The Hidden Markov Model (HMM) of the VQ motif family (PF05678) was downloaded from the Pfam 31.0 database (https://pfam.xfam.org/) and used as query in HMMER3 search, performed in the freeware tool UGENE v1.21^58^. The candidate sequences were confirmed to include the VQ conserved motif using Pfam 31.0 and the NCBI’s Conserved Domain Database (CDD)^59^. Additional domains found were also checked using the Conserved Domain Architecture Retrieval Tool (CDART). Selected features of the *F*. *vesca VQ* (*FvVQ*) genes, protein length, ORF length, molecular weight (kDa) and isoelectric point (PI) of each gene were calculated using ExPASy (https://web.expasy.org/). Nuclear location signals (NLSs) were detected by SeqNLS^60^ and subcellular locations were predicted by LOCALIZER^61^. The chromosomal distribution of the *FvVQ* genes was drawn with Mapchart 2.32^62^ and the intron-exon structure by using the online tool GSDS 2.0^63^. The 1500 bp tracks upstream of *FvVQ*s (or less, if overlapped a previous coding sequence) were screened for known regulatory *cis*-elements and transcription factor binding sites (TFBS) at PLANTCARE and PLANTPAN 2.0 websites. Finally, *the novo* motif discovery was carried out by the MEME Suite v5.0.1 (http://meme-suite.org/tools/meme) on the FvVQ proteins with the following modified parameters: maximum number of motifs set to 20; motif width: 6 to 50.

### Multiple sequence alignment and phylogenetic analysis

Structure-based alignment of *Arabidopsis*, soybean, grapevine and *F*. *vesca* VQ domain sequences was performed in PROMALS3D to gain additional information about the protein secondary structure^64^. This alignment was loaded in MEGA 7.0^65^ as basis to construct unrooted phylogenetic trees by the neighbor-joining (N-J) method with 1000 bootstrap replicates. Evolutionary distances were computed by the p-distance method and pairwise deletion. Orthologous sequences between strawberry and *Arabidopsis* were identified by their best hit in reciprocal BLASTP searches and checked in OrthoMCL DB (http://orthomcl.org/orthomcl/). The homology with *Arabidopsis* proteins, was applied in the construction of a protein functional association network by STRING 10.5 (https://string-db.org/) with medium confidence (0.400).

### Quantitative RT-PCR expression analysis

Specific primer pairs (Supplemental Table S1) were designed by Primer-BLAST (https://www.ncbi.nlm.nih.gov/tools/primer-blast/). Optimal annealing temperatures were assessed by gradient-PCR. Total RNA extraction from strawberry samples, further purification, quality checks, cDNA synthesis, PCR efficiency determination and RT-qPCR runs were carried out as previously described^66^. Reference genes used in this work were *FaEF1α* and *FaACTIN* for tissues and infected fruits, or *FaEF1α* and *FaGAPDH2* for *in vitro* cultured plants^66^. Normalized relative quantification of the gene expression was calculated using the Hellemans’ modification of the Pfaffl method to include several reference genes^67,68^. Gene expression in different tissues was calculated as 2^−ΔCt^ (ΔCt = mean Ct of *VQ* gene minus the geometric mean of reference genes) and represented as heatmap, generated by Heatmapper^69^ with complete linkage and Spearman Rank Correlation settings. A Venn diagram of the up-regulated *FaVQ* for every treatment was generated by BioVenn^70^.

### Statistical analysis

Data were analysed in Microsoft Excel, using the Real Statistics Resource Pack software, release 5.4 (http://www.real-statistics.com/). All data were tested for normality using Shapiro-Wilk test (α = 0.05). One-way ANOVA, followed by Dunnett’s test (two-tailed) post-hoc were performed at α = 0.05 and 0.01. Three biological replicates were used: three fruits sharing the same symptoms or the same developmental stage, or three *in vitro* plantlets form a biological replicate (n = 3). Mean, standard error (SE) and significant differences of *P ≤ 0.05 and **P ≤ 0.01 are represented in figures.

## Supplementary information

Supplemental figures

Supplemental tables

## Data Availability

The datasets generated during the current study are available from the corresponding author on reasonable request.

## References

[1] Garner CM, Kim SH, Spears BJ, Gassmann W (2016). Express yourself: Transcriptional regulation of plant innate immunity. Seminars in Cell & Developmental Biology.

[2] Alves MS (2014). Transcription Factor Functional Protein-Protein Interactions in Plant Defense Responses. Proteomes.

[3] Tsuda K, Somssich IE (2015). Transcriptional networks in plant immunity. New Phytol.

[4] Eulgem T, Rushton PJ, Robatzek S, Somssich IE (2000). The WRKY superfamily of plant transcription factors. Trends Plant Sci.

[5] Jiang J (2017). WRKY transcription factors in plant responses to stresses. J Integr Plant Biol.

[6] Rushton PJ, Somssich IE, Ringler P, Shen QJ (2010). WRKY transcription factors. Trends Plant Sci.

[7] Bakshi M, Oelmüller R (2014). WRKY transcription factors. Plant Signaling & Behavior.

[8] Cheng Y (2012). Structural and functional analysis of VQ motif-containing proteins in Arabidopsis as interacting proteins of WRKY transcription factors. Plant Physiol.

[9] Li N, Li X, Xiao J, Wang S (2014). Comprehensive analysis of VQ motif-containing gene expression in rice defense responses to three pathogens. Plant Cell Reports.

[10] Zhou Y (2016). Structural and Functional Characterization of the VQ Protein Family and VQ Protein Variants from Soybean. Sci Rep.

[11] Wang M (2015). A comprehensive survey of the grapevine VQ gene family and its transcriptional correlation with WRKY proteins. Front Plant Sci.

[12] Zhong Y, Guo C, Chu J, Liu H, Cheng Z-M (2017). Microevolution of the VQ gene family in six species of Fragaria. Genome.

[13] Jing Y, Lin R (2015). The VQ Motif-Containing Protein Family of Plant-Specific Transcriptional Regulators. Plant Physiol.

[14] Jiang SY, Sevugan M, Ramachandran S, Valine-glutamine VQ (2018). motif coding genes are ancient and non-plant-specific with comprehensive expression regulation by various biotic and abiotic stresses. BMC Genomics.

[15] Li Y (2014). MOTIF-CONTAINING PROTEIN29 represses seedling deetiolation by interacting with PHYTOCHROME-INTERACTING FACTOR1. Plant Physiol.

[16] Lai Z (2011). Arabidopsis sigma factor binding proteins are activators of the WRKY33 transcription factor in plant defense. Plant Cell.

[17] Dong Q (2018). Structural and functional analyses of genes encoding VQ proteins in apple. Plant science: an international journal of experimental plant biology.

[18] Wang H, Hu Y, Pan J, Yu D, Arabidopsis VQ (2015). motif-containing proteins VQ12 and VQ29 negatively modulate basal defense against Botrytis cinerea. Sci Rep.

[19] Le Berre JY (2017). Transcriptome dynamic of Arabidopsis roots infected with Phytophthora parasitica identifies VQ29, a gene induced during the penetration and involved in the restriction of infection. PLoS One.

[20] Qiu J-L (2008). Arabidopsis MAP kinase 4 regulates gene expression through transcription factor release in the nucleus. The EMBO Journal.

[21] Pecher P (2014). The Arabidopsis thaliana mitogen-activated protein kinases MPK3 and MPK6 target a subclass of ‘VQ-motif’-containing proteins to regulate immune responses. New Phytol.

[22] Weyhe M, Eschen-Lippold L, Pecher P, Scheel D, Lee J (2014). Ménage à trois. Plant Signaling & Behavior.

[23] DiMeglio LM, Staudt G, Yu H, Davis TM (2014). A Phylogenetic Analysis of the Genus Fragaria (Strawberry) Using Intron-Containing Sequence from the ADH-1 Gene. PLOS ONE.

[24] Schwab, W., Schaart, J. G. & Rosati, C. In *Genetics and Genomics of* Rosaceae (eds Folta, K. M. & Gardiner, S. E.) 457–486 (Springer New York, 2009).

[25] Shulaev V (2011). The genome of woodland strawberry (Fragaria vesca). Nat Genet.

[26] Edger PP (2018). Single-molecule sequencing and optical mapping yields an improved genome of woodland strawberry (Fragaria vesca) with chromosome-scale contiguity. Gigascience.

[27] Tennessen JA, Govindarajulu R, Ashman TL, Liston A (2014). Evolutionary origins and dynamics of octoploid strawberry subgenomes revealed by dense targeted capture linkage maps. Genome Biol Evol.

[28] Darwish O, Shahan R, Liu Z, Slovin JP, Alkharouf NW (2015). Re-annotation of the woodland strawberry (Fragaria vesca) genome. BMC Genomics.

[29] Li Y (2018). Genome re-annotation of the wild strawberry Fragaria vesca using extensive Illumina- and SMRT-based RNA-seq datasets. DNA Research.

[30] Hirakawa H (2014). Dissection of the octoploid strawberry genome by deep sequencing of the genomes of Fragaria species. DNA Res.

[31] Rousseau-Gueutin M (2009). Tracking the evolutionary history of polyploidy in Fragaria L. (strawberry): New insights from phylogenetic analyses of low-copy nuclear genes. Molecular Phylogenetics and Evolution.

[32] Denoyes-Rothan B (2003). Genetic Diversity and Pathogenic Variability Among Isolates of Colletotrichum Species from Strawberry. Phytopathology.

[33] Amil-Ruiz F (2016). Partial Activation of SA- and JA-Defensive Pathways in Strawberry upon Colletotrichum acutatum Interaction. Front Plant Sci.

[34] Guidarelli M (2011). Colletotrichum acutatum interactions with unripe and ripe strawberry fruits and differential responses at histological and transcriptional levels. Plant Pathology.

[35] Wang F (2017). Comparative Transcriptomics Reveals Differential Gene Expression Related to Colletotrichum gloeosporioides Resistance in the Octoploid Strawberry. Front Plant Sci.

[36] Kim DY (2013). Expression analysis of rice VQ genes in response to biotic and abiotic stresses. Gene.

[37] Rinerson CI, Rabara RC, Tripathi P, Shen QJ, Rushton PJ (2015). The evolution of WRKY transcription factors. BMC Plant Biology.

[38] Wang Y (2017). Genome-wide analysis of VQ motif-containing proteins in Moso bamboo (Phyllostachys edulis). Planta.

[39] Zhang G (2015). Genome-Wide Identification and Analysis of the VQ Motif-Containing Protein Family in Chinese Cabbage (Brassica rapa L. ssp. Pekinensis). Int J Mol Sci.

[40] Guo J (2018). Identification, characterization and expression analysis of the VQ motif-containing gene family in tea plant (Camellia sinensis). BMC genomics.

[41] Chu, W. *et al*. Genome-wide analysis of poplar VQ gene family and expression profiling under PEG, NaCl, and SA treatments. *Tree Genetics & Genomes* **12**, 10.1007/s11295-016-1082-z (2016).

[42] Kallberg M (2012). Template-based protein structure modeling using the RaptorX web server. Nat Protoc.

[43] Wright PE, Dyson HJ (2015). Intrinsically Disordered Proteins in Cellular Signaling and Regulation. Nature reviews. Molecular cell biology.

[44] Pazos, F., Pietrosemoli, N., García-Martín, J. & Solano, R. Protein intrinsic disorder in plants. *Frontiers in Plant Science* **4**, 10.3389/fpls.2013.00363 (2013). 10.3389/fpls.2013.00363 PMC3770944 24062761

[45] Csukasi F (2011). Gibberellin biosynthesis and signalling during development of the strawberry receptacle. New Phytologist.

[46] Symons GM (2012). Hormonal changes during non-climacteric ripening in strawberry. Journal of Experimental Botany.

[47] Watson R, Wright CJ, McBurney T, Taylor AJ, Linforth RST (2002). Influence of harvest date and light integral on the development of strawberry flavour compounds. Journal of Experimental Botany.

[48] van Verk MC, Pappaioannou D, Neeleman L, Bol JF, Linthorst HJM (2008). A Novel WRKY Transcription Factor Is Required for Induction of PR-1a Gene Expression by Salicylic Acid and Bacterial Elicitors. Plant Physiology.

[49] Machens F, Becker M, Umrath F, Hehl R (2014). Identification of a novel type of WRKY transcription factor binding site in elicitor-responsive cis-sequences from Arabidopsis thaliana. Plant Molecular Biology.

[50] Birkenbihl RP, Diezel C, Somssich IE (2012). Arabidopsis WRKY33 Is a Key Transcriptional Regulator of Hormonal and Metabolic Responses toward Botrytis cinerea Infection. Plant Physiology.

[51] Zheng Z, Qamar SA, Chen Z, Mengiste T (2006). Arabidopsis WRKY33 transcription factor is required for resistance to necrotrophic fungal pathogens. The Plant Journal.

[52] Hu P (2013). JAV1 controls jasmonate-regulated plant defense. Mol Cell.

[53] Yan C (2018). Injury Activates Ca(2+)/Calmodulin-Dependent Phosphorylation of JAV1-JAZ8-WRKY51 Complex for Jasmonate Biosynthesis. Mol Cell.

[54] Luhua S, Ciftci-Yilmaz S, Harper J, Cushman J, Mittler R (2008). Enhanced Tolerance to Oxidative Stress in Transgenic Arabidopsis Plants Expressing Proteins of Unknown Function. Plant Physiology.

[55] Chen, J. *et al*. Arabidopsis VQ10 interacts with WRKY8 to modulate basal defense against Botrytis cinerea. *J Integr Plant Biol*, 10.1111/jipb.12664 (2018). 10.1111/jipb.12664 29727045

[56] Encinas-Villarejo S (2009). Evidence for a positive regulatory role of strawberry (Fragaria × ananassa) Fa WRKY1 and Arabidopsis At WRKY75 proteins in resistance. Journal of Experimental Botany.

[57] Margara, J. *Bases de la multiplication végétative: les méristèmes et l’organogenèse*. (Paris: Institut national de la recherche agronomique, 1989).

[58] Okonechnikov K, Golosova O, Fursov M (2012). Unipro UGENE: a unified bioinformatics toolkit. Bioinformatics (Oxford, England).

[59] Marchler-Bauer A (2017). CDD/SPARCLE: functional classification of proteins via subfamily domain architectures. Nucleic Acids Research.

[60] Lin J-r, Hu J (2013). SeqNLS: Nuclear Localization Signal Prediction Based on Frequent Pattern Mining and Linear Motif Scoring. PLoS ONE.

[61] Sperschneider J (2017). LOCALIZER: subcellular localization prediction of both plant and effector proteins in the plant cell. Scientific Reports.

[62] Voorrips RE (2002). MapChart: Software for the Graphical Presentation of Linkage Maps and QTLs. Journal of Heredity.

[63] Hu B (2015). GSDS 2.0: an upgraded gene feature visualization server. Bioinformatics (Oxford, England).

[64] Pei J, Kim B-H, Grishin NV (2008). PROMALS3D: a tool for multiple protein sequence and structure alignments. Nucleic Acids Research.

[65] Kumar S, Stecher G, Tamura K (2016). MEGA7: Molecular Evolutionary Genetics Analysis Version 7.0 for Bigger Datasets. Molecular biology and evolution.

[66] Amil-Ruiz F (2013). Identification and validation of reference genes for transcript normalization in strawberry (Fragaria x ananassa) defense responses. PLoS One.

[67] Hellemans J, Mortier G, De Paepe A, Speleman F, Vandesompele J (2007). qBase relative quantification framework and software for management and automated analysis of real-time quantitative PCR data. Genome Biology.

[68] Pfaffl MW (2001). A new mathematical model for relative quantification in real-time RT–PCR. Nucleic Acids Research.

[69] Babicki S (2016). Heatmapper: web-enabled heat mapping for all. Nucleic Acids Research.

[70] Hulsen T, de Vlieg J, Alkema W (2008). BioVenn - a web application for the comparison and visualization of biological lists using area-proportional Venn diagrams. BMC Genomics.

