# Supplementary material for: The VQ motif-containing proteins in the diploid and octoploid strawberry
Source: Sci Rep. 2019 Mar 20;9:4942. doi: 10.1038/s41598-019-41210-4 (PMC6427031; doi:10.1038/s41598-019-41210-4)
Supplement: Supplementary file 1 — Supplemental figures [file 41598_2019_41210_MOESM1_ESM.pdf]

SUPPLEMENTARY FIGURES

The VQ motif-containing proteins in the diploid and octoploid strawberry.

José Garrido-Gala[1], José Javier Higuera[1], Juan Muñoz-Blanco[1], Francisco Amil-Ruiz[2] and José L. Caballero[1*]

[1]Departamento de Bioquímica y Biología Molecular, Edificio Severo Ochoa-C6, Campus Universitario de Rabanales y Campus de Excelencia Internacional Agroalimentario CEIA3, Universidad de Córdoba, 14071 Córdoba, Spain.

[2]Unidad de Bioinformática, Servicio Central de Apoyo a la Investigación (SCAI), Universidad de Córdoba, 14071 Córdoba, Spain.

*** Correspondence:**
José L. Caballero
bb1carej@uco.es

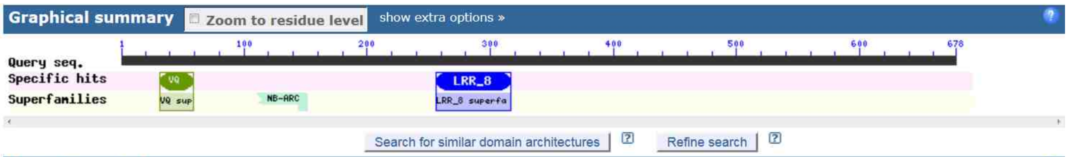

List of domain hits

Name	Accession	Description	Interval	E-value
[+] LRR_8	pfam13855	Leucine rich repeat;	256-317	3.69e-08
[+] NB-ARC super family	cl26397	NB-ARC domain;	110-151	3.12e-05
[+] VQ	pfam05678	VQ motif; This short motif is found in a variety of plant proteins. These proteins vary ...	32-59	9.84e-05

References:

Marchler-Bauer A et al. (2017), *"CDD/SPARCLE: functional classification of proteins via subfamily domain architectures."*, **Nucleic Acids Res.45**(D)200-3.

Marchler-Bauer A et al. (2015), *"CDD: NCBI's conserved domain database."*, **Nucleic Acids Res.43**(D)222-6.

Marchler-Bauer A et al. (2011), *"CDD: a Conserved Domain Database for the functional annotation of proteins."*, **Nucleic Acids Res.39**(D)225-9.

Marchler-Bauer A, Bryant SH (2004), *"CD-Search: protein domain annotations on the fly."*, **Nucleic Acids Res.32**(W)327-331.

Taxa	Taxid	Sequences	Accessions	Domain Names
Streptophyta	35493	4271	pfam05678	VQ
Spermatophyta	58024	59	pfam05678; cl26464	VQ; Atrophin-1
Pentapetalae	1437201	19	pfam05678; cl25764	VQ; PAT1
Pentapetalae	1437201	13	pfam05678; cl27891	VQ; LRR_3
Mesangiospermae	1437183	10	cl26464; pfam05678	Atrophin-1; VQ
rosids	71275	9	pfam05678; cl25887	VQ; RRM
Mesangiospermae	1437183	8	pfam05678; pfam00931 ;cl25992	VQ; NB-ARC ;LRR_8
Brassicaceae	3700	7	pfam07887; pfam05678 ;cl27891	Calmodulin_bind; VQ ;LRR_3
Brassicaceae	3700	5	pfam05678; cl17169	VQ; RRM_SF
Brassica	3705	5	pfam01049; pfam05678	Cadherin_C; VQ
rosids	71275	5	pfam05678; cl28087	VQ; FtsK
Pentapetalae	1437201	4	pfam05678; pfam00931	VQ; NB-ARC
Maleae	721813	3	pfam05678; pfam14723	VQ; SSFA2_C
Oryza sativa	4530	3	cl23735; pfam05678	H4; VQ ;
rosids	71275	3	pfam05678; cl27489	VQ; Glucosaminidase
Brassica	3705	2	pfam05678; pfam11383	VQ; DUF3187
Brassica oleracea var. oleracea	109376	2	pfam05678; cl00031	VQ; ALBUMIN
Embryophyta	3193	2	cl26386; pfam05678	DNA_pol3_gamma3; VQ
Brassica	3705	2	pfam05678; cl27863	VQ; SSL_OB
rosids	71275	2	pfam05678; cl26247	VQ; DNA_pol3_delta2
Erythranthe guttata	4155	2	pfam05678; cl25836	VQ; Retinal
malvids	91836	2	pfam05678; cl25764 ;cl26464	VQ; PAT1 ;Atrophin-1
Arachis	3817	2	pfam05678; cl11531	VQ; ZapB
Pentapetalae	1437201	2	pfam05678; cl26386	VQ; DNA_pol3_gamma3
Populus euphratica	75702	1	pfam05678; pfam13266	VQ; DUF4057

Accesion	Description
XP_015612015.1 GI:1002297180	PREDICTED: putative disease resistance protein RGA3 [Oryza sativa Japonica Group], 1137 aa protein
XP_010673130.1 GI:731324745	PREDICTED: putative disease resistance protein RGA3 [Beta vulgaris subsp. vulgaris], 679 aa protein
BAT07727.1 GI:937932812	Os09g0365000 [Oryza sativa Japonica Group], 1079 aa protein
ALO70146.1 GI:953415386	NBS-LRR-like resistance protein [Oryza sativa], 1137 aa protein
ALO70147.1 GI:953415388	NBS-LRR-like resistance protein [Oryza sativa], 992 aa protein
ALO70148.1 GI:953415390	NBS-LRR-like resistance protein [Oryza sativa], 1107 aa protein
ALO70149.1 GI:953415392	NBS-LRR-like resistance protein [Oryza sativa], 1137 aa protein
XP_021745766.1 GI:1219164272	putative disease resistance protein RGA3 [Chenopodium quinoa], 511 aa protein

Supplemental Figure S1. Protein domains of strawberry VQ13 found by the Conserved Domain Database (CDD) with tables showing the diversity of VQ protein architectures found along different taxa, and examples of proteins with the same domains found in strawberry VQ13 retrieved by CDART.

```
                                               *       * *     *
GmVQ64     V I I N T - - - Q Y V E - T D A T S F K S V V Q K L T G - - K D S D I
GmVQ37     V I I N T - - - Q Y V E - T D A T S F K S V V Q K L T G - - K D S D N
FvVQ8      V I I N T - - - K Y V E - T D A M S F K D V V Q K M T G - - K D S R V
AtVQ10     V F I N T - - - Q Y V E - T D A R S F K T V V Q E L T G - - K N A I V
AtVQ1      V F I N T - - - Q Y V Q - T D A R S F K T I V Q E L T G - - K N A V V
GmVQ55     V Q I E T - - - R Y V Q - T D A A N F R D V V Q S L T G - - K N S S T
GmVQ33     V H I E T - - - R Y V E - T D A I N F R D V V Q H L T G - - K N S S T
GmVQ17     V H I E T - - - R Y V E - T D A I H F R D V V Q H L T G - - K N S S T
FvVQ16     K Y I S S P - - M M V Q A N N A S E F R A I V Q E L T G - - Q N S D A
GmVQ7      V Y I S N P - - M K I K - T S A S E F R A L V Q E L T G - - Q D A E S
GmVQ70     V Y I S N P - - M K I K - T S A S E F R A L V Q E L T G - - Q D A E S
FvVQ18     V Y I S N P - - M K V T - A S A S E F R A L V Q Q L T G - - Q D A D D
VvVQ6      V Y I G N P - - R M V K - V K E S E F R A L V Q E L T G - - Q D A D I
AtVQ16     R Y I S N P - - M R V E - T C P S K F R E L V Q E L T G - - Q D A A D
AtVQ23     R Y I S N P - - M R V Q - T C A S K F R E L V Q E L T G - - Q D A V D
FvVQ25     V Y I S S P - - M K V Q - T S A S K F R A L V Q E L T G - - R D S D A
VvVQ10     V Y I S S P - - M K V K - T S A S K F R A L V Q E L T G - - R D S D V
VvVQ12     T Y I S S P - - M K V K A S S A S E F R A I V Q E L T G - - C N S N P
GmVQ12     T Y I S S P - - M K V K - T S A S N F R A L V Q E L T G - - Q A S N V
GmVQ54     T Y I S S P - - V K V K - T S A S N F R A L V Q E L T G - - Q Y S N V
GmVQ63     T Y I S S P - - V K V K - T S A S N F R A L V Q E L T G - - Q Y S N V
GmVQ72     T Y I S S P - - V L V R A Y D A S E F R S V V Q Q L T G - - N D S N S
GmVQ9      T Y I S N P - - V L V R A C D A S E F R S V V Q Q L T G - - K D T N K
GmVQ35     I Y A V S P - - K V L H - V P A G D F M N V V Q R L T G - - P S S G D
GmVQ19     I Y T V S P - - K V L H - V T V S D F M N V V Q R L T G - - P S S G A
GmVQ25     I Y S V S P - - K V I H - V T P G D F M D V V Q R L T G - - A S I G E
GmVQ13     I Y S V S P - - K V I H - V T P G D F M D V V Q R L T G - - A S S G E
AtVQ21     I Y A V S P - - K V V H - A T A S E F M N V V Q R L T G - - I S S G V
GmVQ71     V H L K S P - - K V I H - V R P E E F M S L V Q Q L T G - - N P V S A
GmVQ8      V H L K S P - - K V I H - V R P E E F M S L V Q Q L T R - - N P V S A
FvVQ19     V Y L I S P - - R I I H - V Q P E E F M A L V Q R L T G - - N H E S S
AtVQ3      I Y T V S P - - R I I H - T H P N N F M T L V Q R L T G - - K T S T S
AtVQ2      I Y T V T P - - R I I H - T H P N N F M T L V Q R L T G - - Q T S T S
GmVQ45     I Y T H S P - - K V I H - T Q P K D F M S L V Q K L T G - - L S R S G
GmVQ28     I Y T H S P - - K V I H - T Q P K D F M S L V Q K L T G - - L S R S D
VvVQ2      I Y T H S P - - K I I H - T Q A R D F M A L V Q K L T G - - L S S S S
FvVQ12     I Y T H S P - - K I I H - T H P R D F M A L V Q K L T G - - L S R F E
FvVQ11     I Y T Q S P - - K V I H - T R P H D F M A L V Q K L T G - - L S P S D
AtVQ20     I Y T H T P - - R I I H - T N P K D F M A L V Q K L T G - - M T H S D
GmVQ31     I Y T H P P - - K V I H - T H A R N F M E L V Q K L T G - - L Y R T D
AtVQ8      I Y A H S P - - K V I H - T R A E D F M A L V Q R L T G L D E I I R R
GmVQ74     I Y T E S P - - K V I H - T K A K D F M A L V Q R L T G - - R S S T N
GmVQ46     I Y T E S P - - K I I H - T K A K D F M A L V Q R L T G - - R S S S T
FvVQ3      I Y T V S P - - K V I H - A S V N E F M S L V Q R L T G - - P S S S P
VvVQ15     I Y T V S P - - K I I H - T Q P S E F M T L V Q R L T G - - L S S S S
FvVQ5      I Y T V S P - - K I I H - T N P S D F M N L V Q R L T G - - L P P S N
GmVQ59     I Y T V S P - - K V I H - T T P S D F M S L V Q R L T G - - S S S S S
GmVQ51     I Y T V S P - - K V I H - T T P S D F M S L V Q R L T S - - S S S S S
GmVQ24     I Y T V S P - - K V I H - T T P S D F M N L V Q R L T G - - S S S S S
GmVQ14     I Y T V S P - - K V I H - T T P S D F M N L V Q R L T G - - S S S S S
GmVQ36     I H V Y T P - - E I I K - T D A A N F R E L V Q R L T G - - K P K E E
GmVQ65     I H V Y A P - - E I I K - T D A A N F K E L V Q R L T G - - K P K E E
VvVQ11     I H V F A P - - E I I Q - T D A A N F R D L V Q R L T G - - K P A A N
AtVQ18     I H I F A P - - E V I K - T D V K N F R S L V Q S L T G - - K P A P G
AtVQ26     I H I F A P - - E I I N - T D V K N F R T L V Q S L T G - - K P E I T
FvVQ20     I H I F A P - - E I I K - T D V A N F R E L V Q R L T G - - K P S D N
AtVQ25     I H I F A P - - E I I K - T D V A N F R E L V Q S L T G - - K P D D Q
AtVQ17     I H I F A P - - E I I K - T D V A N F R E I V Q N L T G - - K Q D H H
VvVQ7      I H I F A P - - E I I K - T D V E N F R E L V Q R L T G - - K P S A A
GmVQ4      I H I F A P - - E I I K - T D V E N F R E L V Q K L T G - - R P S G E
GmVQ44     I H I F A P - - E I I K - T D V E N F R E L V Q K L T G - - K P S G E
GmVQ42     I K V L R P - - K V Y I - T D S S S F K K L V Q E L T G - - N G S S N
GmVQ68     I K I L R P - - K V Y I - T D S S S F K K L V Q E L T G - - N G S P T
VvVQ9      I K V L R P - - K V Y I - T D S S S F K K L V Q E L T G - - N G T S V
FvVQ22     I R L L R P - - K V Y I - T D C S S F K T L V Q D L T G - - N G S S N
FvVQ23     I R L L R P - - K V Y I - T D C S S F K T L V Q D L T G - - N G S S N
AtVQ5      - - - - - - - - Q V Y I - I D K N D F K S L V Q Q L T S - - P Q P C D
AtVQ6      S P L P Q P - - Q G Y S - M S N N D F T S I V Q Q L T D - - S P S R E
AtVQ32     - - - - - - - - L V Y N - I N K T D F R S I V Q Q L T G - - L G S T S
VvVQ14     Q Q Q H Q P - - P V Y N - I N K N D F R D V V Q K L T G - - S P A H E
GmVQ10     N L Q H Q P - - P V Y N - I N K N D F R D V V Q K L T G - - S P A H D
FvVQ14     P N Q H Q P - - P V Y N - I N K N D F R D V V Q K L T G - - S P A H T
AtVQ9      L H Q H Q P - - P V Y N - I N K N D F R D V V Q K L T G - - S P A H E
GmVQ21     Q V Q H Q P - - P V Y N - I N K N D F R D V V Q K L T G - - S P A H D
GmVQ5      P S E S Q P - - Q V Y N - I S K N D F R D M V Q K L T G - - S P G H T
AtVQ14     R L Q T Q P - - Q V Y N - I S K N D F R S I V Q Q L T G - - S P S R E
FvVQ10     R Q Q P Q P - - Q V Y N - I S K N D F R N I V Q Q L T G - - S P S Q D
GmVQ1      R Q Q P Q P - - Q V Y N - I S K N D F R D I V Q Q L T G - - S P S Q D
VvVQ3      R Q Q P Q P - - Q V Y N - I S K N D F R N I V Q Q L T G - - S P S Q E
```

GmVQ2	Q Q Q P Q P - - Q V Y N - I S K N E F R D I V Q K L T G - - S P S Q D
GmVQ40	Q P Q P Q P - - Q V Y N - I S K N D F R D I V Q Q L T G - - S P S Q S
GmVQ39	Q Q Q P Q P - - Q V Y N - I S K N D F R D I V Q Q L T G - - S P S Q S
GmVQ41	Q Q Q P Q P - - Q V Y N - I S K N D F R D I V Q Q L T G - - S P S Q S
AtVQ29	L N P M H P - - H V Y R - V E P V N F K V L V Q R L T G - - A P E H E
AtVQ12	P Q R M H P - - K V Y R - V E P V N F K E L V Q R L T G - - A E D V E
VvVQ18	L P P T P P - - R V Y K - V E S A N F R E V V Q Q L T G - - S P E F Q
GmVQ66	M P P T P V - - K V Y K - V D A I N F R E V V Q Q L T G - - A P K H K
GmVQ15	M P P T P I - - K V Y K - V D A I N F R D V V Q Q L T G - - A P E H E
FvVQ1	L P P T P P - - R V Y K - V D P I N F R D L V Q K L T S - - A P E Y Q
GmVQ47	P A P T P I - - R V Y K - V D A I N F R D L V Q Q L T G - - A P E F K
GmVQ3	P A P T P I - - R V Y K - V D A I N F R D L V Q Q L T G - - A P E F K
GmVQ56	- - S Y P T - - T F V Q - A D T T S F K Q V V Q M L T G - - S S E T A
GmVQ61	- - P Y P T - - T F V Q - A D T S S F K Q V V Q M L T G - - S S E T A
FvVQ6	- - - Y P T - - T F V Q - A D T N S F K Q V V Q M L T G - - S S E T A
AtVQ4	- - P Y P T - - T F V Q - A D T S S F K Q V V Q M L T G - - S A E R P
GmVQ29	- - P Y P T - - T F V Q - A D T T S F K Q V V Q M L T G - - S T Q T A
GmVQ53	- - P Y P T - - T F V Q - A D T N S F K Q V V Q M L T G - - S T Q T A
FvVQ9	- - - Y A T - - T F V Q - A D S S N F K H V V Q M L T G - - S S E T T
GmVQ26	N P Y P T - - - T F V Q - A D T S T F K H V V Q M L T G - - S S E T T
GmVQ49	- - P Y P T - - T F V Q - A D T S T F K Q V V Q M L T G - - S S E T T
GmVQ50	- - - Y P T - - T F V Q - A D T S T F K Q V V Q M L T G - - S S D T T
GmVQ57	- - - Y P T - - T F V Q - A D T S T F K Q V V Q M L T G - - S S D T T
AtVQ33	N P Y P T - - - T F V Q - A D T S T F K Q V V Q M L T G - - S S T D T
AtVQ19	T - - - - - - - T F V Q - A D S S S F K Q V V Q M L T G - - S S S P R
VvVQ16	- N P Y P T - - T F V Q - A D A N S F K Q V V Q R L T G - - S S K P T
AtVQ13	D M Y E T - - - T F I R - T D P S S F K Q V V Q L L T G - - I P K N P
GmVQ20	- - K P L T - - T F V Q - T N S D A F R D V V Q R L T G - - P S E A S
GmVQ30	- - K P L T - - T F V Q - T N S D A F R E V V Q R L T G - - P S E A S
AtVQ31	T C K P V T - - T F V Q - T D T N T F R E I V Q R L T G - - P T E N N
FvVQ2	D C K P L T - - T F V H - A D T S T F Q K V V Q R L T G - - A G P N Q
VvVQ4	P P T S T - - - T F V Q - A D A T T F R D L V Q K L T G - - A A V D S
AtVQ11	S Y A T E P N T M F V Q - A D P S N F R N I V Q K L T G - - A P P D I
FvVQ21	- - - P N T - - T Y V Q - A D P S S F R A V V Q K L T G - - A T E D P
VvVQ8	- - - N N T - - T F V Q - A D P S N F R A V V Q H L T G - - A S P D S
GmVQ27	- - T P N T - - T F V Q - A N P S N F R A V V Q K L T G - - A S D D P
GmVQ38	- - T P N T - - T F V Q - A D P S N F R A V V Q K L T G - - A S D D P
GmVQ67	S R R A P T - - T V L T - T D T T N F R A M V Q E F T G - - I P A P P
GmVQ48	S R R A P T - - T V L T - T D T T N F R A M V Q E F T G - - I P A P P
GmVQ18	S R R A P T - - T V L T - T D T T N F R A M V Q E F T G - - I P A P P
FvVQ4	S R R A P T - - T V L T - T D T T N F R A M V Q E F T G - - I P A P P
GmVQ34	S R R A P T - - T V L T - T D T T N F R A M V Q E F T G - - I P A Q P
VvVQ17	S R R A P T - - T V L T - T D T T N F R A M V Q E F T G - - I P A Q P
GmVQ23	S R R A P T - - T V L T - T D T N N F R S M V Q E F T G - - I S A P P
GmVQ22	S R R A P T - - - - - - - T D T N N F R S M V Q E F T G - - I P A P P
GmVQ11	S R R A P T - - T V L T - T D T N N F R S M V Q E F T G - - I P A P P
AtVQ30	S R R A P T - - T V L T - T D T S N F R A M V Q E F T G - - I P A P P
AtVQ7	S R R A P T - - T V L T - T D T S N F R A M V Q E F T G - - V P A S P
FvVQ15	S R R A P T - - T V L T - T D T S N F R A M V Q E F T G - - I P A P P
GmVQ60	S R R A P T - - T V L T - T D T S N F R A M V Q E F T G - - I P A P P
VvVQ13	S R R A P T - - T V L T - T D T S N F R A M V Q E F T G - - I P A P P
AtVQ34	S R R A P T - - T V L T - T D T S N F R A M V Q E F T G - - N P S T P
FvVQ24	S R R T P T - - T L L N - T D T A N F R A M V Q Q F T G - - G P S T A
GmVQ58	S R R T P T - - T L L N - T D T S N F R A M V Q Q F T G - - A P S A P
AtVQ22	S R R T P T - - T L L N - T D T S N F R A M V Q Q Y T G - - G P S A M
AtVQ27	S R R T P T - - T L F N - T D T A N F R A M V Q Q F T G - - G P S A V
GmVQ73	S R R T P T - - T L L N - T D T T N F R A M V Q Q F T G - - G P S A P
GmVQ52	S R R T P T - - T L L N - T D T T N F R A M V Q Q F T G - - G P S A P
AtVQ28	S R R A I P - T T L L N - A N P S N F R A L V Q K F T G - - R S A G G
VvVQ1	P R R I P A - - T L L T - A N T T N F R A L V Q Q F T G - - R P T T P
GmVQ43	S K R T P T - - T L L N - A N P T N F R A L V Q Q F T G - - C P R T T
GmVQ32	S K S T P I - - T L L K - A N T S N F R A L V Q Q F T G - - C P T T T
GmVQ16	S K S T P I - - T L L K - A N T S N F R A L V Q Q F T G - - C P T T T
GmVQ62	S K K T P T - - T L L N - A N T T N F R A L V Q Q F T G - - C H S T T
FvVQ7	S K K T P I - - T L L N - A N A N N F R A L V Q Q F T G - - C A A S S
AtVQ24	S N K K S Q - T T F I T - A D A A N F R Q M V Q Q V T G - - A K F L G
AtVQ15	S K K S Q T - - T F I T - A D P S N F R Q M V Q Q V T G - - A K Y I D
FvVQ17	S K R T Q T - - T F I T - A D P A N F R Q M V Q Q V T G - - V R F S G
VvVQ5	S K R S Q T - - T F I T - A D P A N F R Q M V Q Q V T G - - V R F G N
GmVQ69	S K R S Q T - - T F I T - A D P A N F R Q M V Q Q V T G - - V R F G G
GmVQ6	S K R S Q T - - T F I T - A D P A N F R Q M V Q Q V T G - - V R F G G
FvVQ13	K K R R A R - T A V Y T - V E T S E F R S L V Q S L T G - - P S R S P
	upstream motif … core motif
Consensus aa	p h h p h s s s s F + x I V Q p L T G
Consensus structure	E E E E H H H H H H H H H H H H

Supplemental Figure S2. VQ domain alignment corresponding to the phylogenetic tree shown in the main text (Fig.2). The same colors to sort the seven different clades obtained and symbols are applicable.

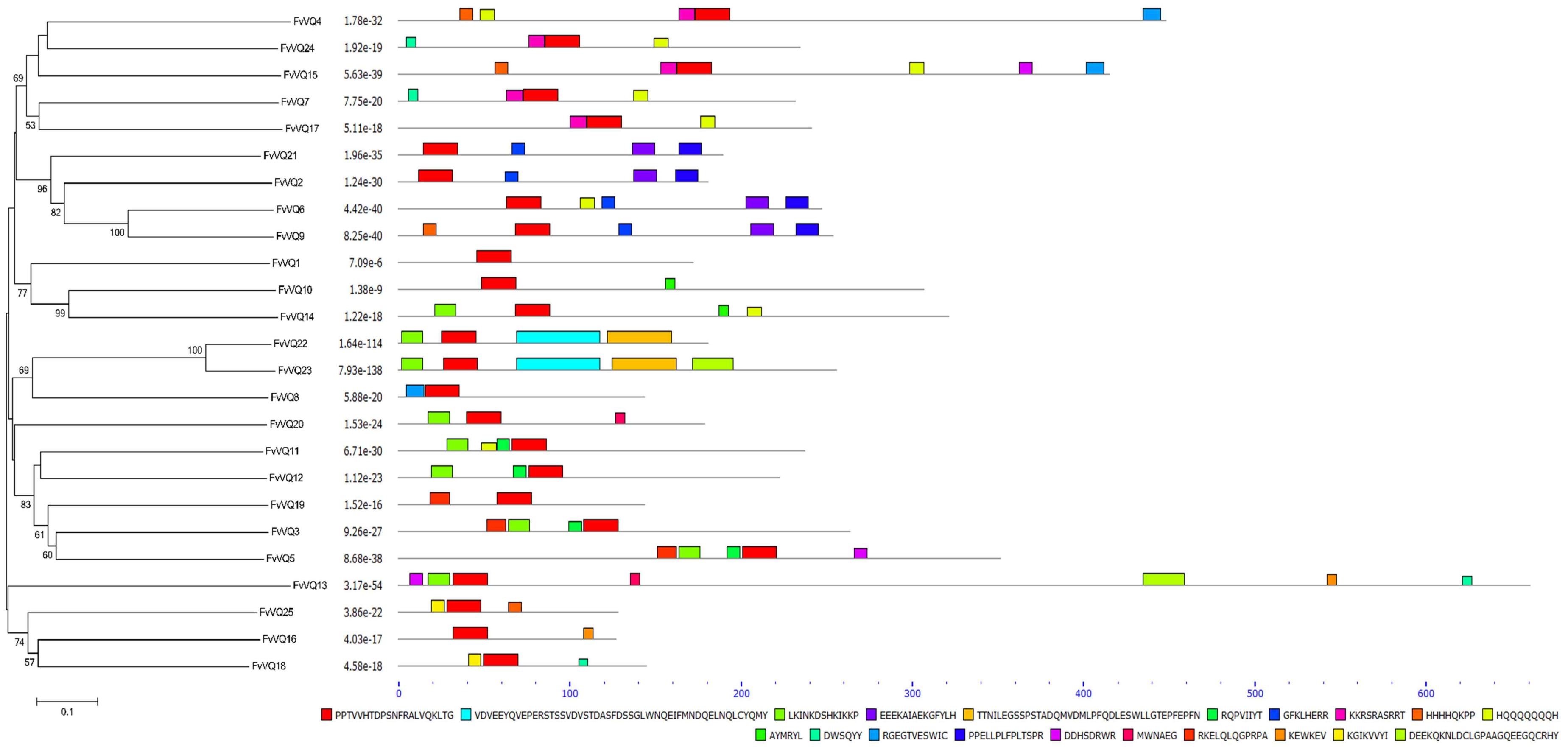

Supplemental Figure S3. Phylogenetic tree of strawberry VQ proteins and motifs found by MEME. The full FvVQ sequences were aligned by MUSCLE and an unrooted tree was constructed using MEGA 7.014 by the neighbour-joining method (1000 bootstrap replicates). The 20 motifs found are schematized in different colors and the combined mach p-values are shown (see Supplemental Table S3 for further details).

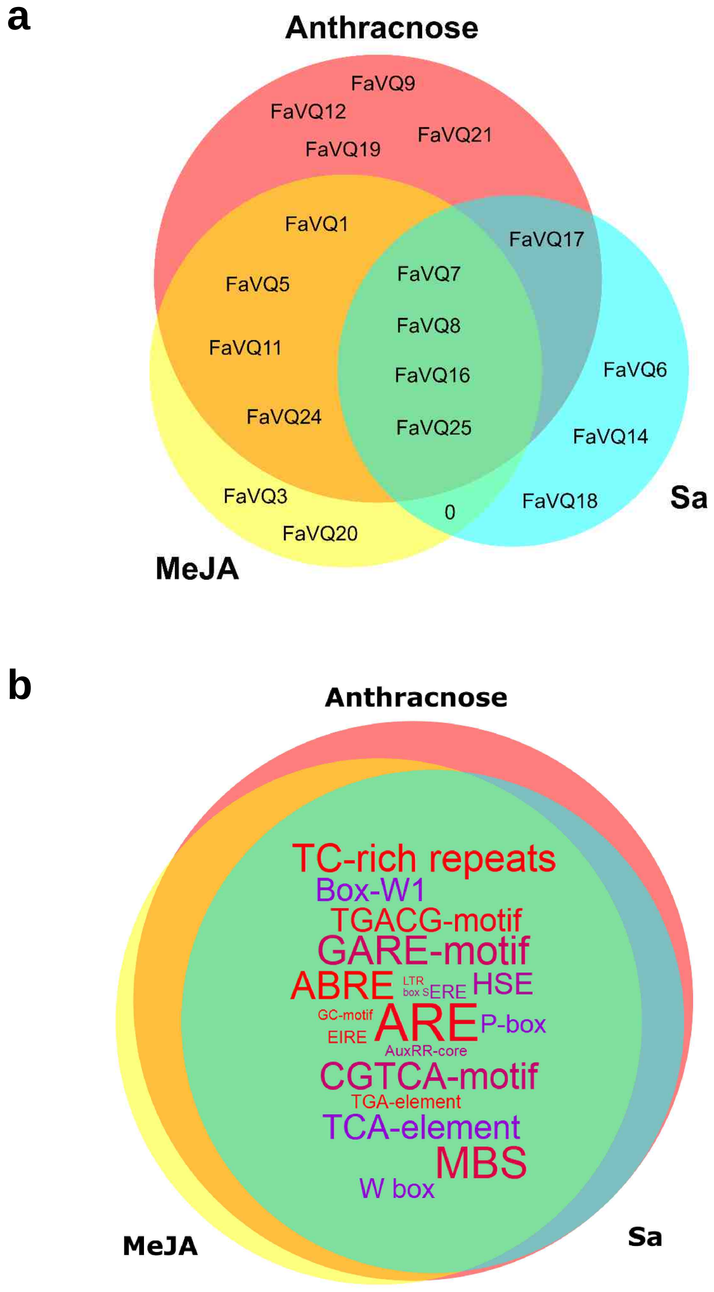

Supplemental Figure S4. (a) Venn diagrams of up-regulated *FaVQs* revealing the unique and common genes under the different treatments shown in Fig. 5-7. **(b)** Venn diagram of the *cis*-regulatory elements present in the up-regulated *FaVQ* genes (by homology with their *FvVQ* orthologs). Only the functional categories “Phytohormone responsiveness” and “Elicitor and stress responses” were used (see Suppl. Table S5). A word cloud was generated, representing the common regulatory sequences (19 out of 27) shared among the up-regulated genes by treatment. Font sizes are proportional to the frequency of the different elements.
